# SABRE hyperpolarisation of vitamin B3 as a function of pH†
†Electronic supplementary information (ESI) available. See DOI: 10.1039/c6sc04043h



**DOI:** 10.1039/c6sc04043h

**Published:** 2016-12-07

**Authors:** A. M. Olaru, M. J. Burns, G. G. R. Green, S. B. Duckett

**Affiliations:** a Centre for Hyperpolarisation in Magnetic Resonance , Department of Chemistry , University of York , YO10 5NY , York , UK . Email: simon.duckett@york.ac.uk; b York Neuroimaging Centre , University of York , YO10 5NY , York , UK

## Abstract

In this work we describe how the signal enhancements obtained through the SABRE process in methanol-*d*_4_ solution are significantly affected by pH. Nicotinic acid (vitamin B3, **NA**) is used as the agent, and changing pH is shown to modify the level of polarisation transfer by over an order of magnitude, with significant improvements being seen in terms of the signal amplitude and relaxation rate at high pH values. These observations reveal that manipulating pH to improve SABRE enhancements levels may improve the potential of this method to quantify low concentrations of analytes in mixtures. ^1^H NMR spectroscopy results link this change to the form of the SABRE catalyst, which changes with pH, resulting in dramatic changes in the magnitude of the ligand exchange rates. The presented data also uses the fact that the chemical shifts of the nicotinic acids NMR resonances are affected by pH to establish that hyperpolarised ^1^H-based pH mapping with SABRE is possible. Moreover, the strong polarisation transfer field dependence shown in the amplitudes of the associated higher order longitudinal terms offers significant opportunities for the rapid detection of hyperpolarised **NA** in H_2_O itself without solvent suppression. ^1^H and ^13^C MRI images of hyperpolarised vitamin B3 in a series of test phantoms are presented that show pH dependent intensity and contrast. This study therefore establishes that when the pH sensitivity of **NA** is combined with the increase in signal gain provided for by SABRE hyperpolarisation, a versatile pH probe results.

## Abstract 2

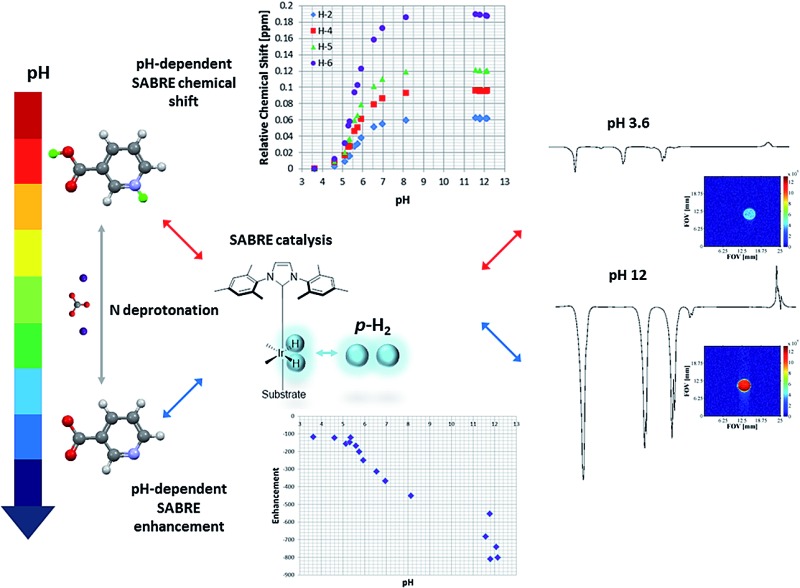
NMR sensitivity enhanced through SABRE hyperpolarisation and pH manipulation enables the use of vitamin B3 as a pH probe.

## 1. Introduction

Magnetic resonance imaging (MRI) is currently one of the most powerful medical diagnostic methods. While it benefits from being non-invasive and extremely versatile in terms of its applicability through harnessing structural, diffusion and flow differences, its lack of sensitivity has, until recently, severely limited its capabilities. This hurdle can be overcome by the use of hyperpolarisation methods, which transfer magnetisation to the nucleus of interest thereby dramatically increasing the detected response.

Besides spin exchange optical pumping,^1^ which enables the diagnosis of pulmonary pathologies using hyperpolarised noble gases,^2–4^ the most advanced technique used in preclinical and clinical MRI investigations is Dynamic Nuclear Polarisation (DNP). In the case of DNP, the sample (containing a free radical and a ^13^C-labelled molecule in a glassing matrix) is cooled down to ∼1 K and electron based polarisation is transferred to the nucleus (typically ^13^C or ^15^N) by microwave irradiation.^5^ *para*-Hydrogen Induced Polarisation (PHIP) is another hyperpolarisation method, which has become increasingly popular due to its efficiency and low cost.^6^ In PHIP signal enhancement is traditionally achieved by a catalysed hydrogenation reaction in which *para*-H_2_ (*p*-H_2_) is used instead of *ortho*-H_2_.^7,8^ Symmetry breakdown in the product leads to hyperpolarisation access which is not just limited to protons as hetero nuclei signals can also be substantially enhanced. A newer approach to polarisation transfer from *p*-H_2_, Signal Amplification by Reversible Exchange (SABRE^9^) allows the substrate to remain chemically unchanged and, instead, achieves magnetisation transfer through the scalar coupling framework of a metal complex. When the binding of both the ligand and H_2_ to this complex are reversible, SABRE allows for the repeated or continuous polarisation of the associated target molecule.^10^

DNP has proven to be capable of hyperpolarising a range of small, biocompatible molecules, such as pyruvate, bicarbonate or ascorbic acid and, in conjunction with localized MR spectroscopy methods, their detection has enabled the real time monitoring of metabolic processes, perfusion, inflammation, as well as the diagnosis and assessment of the response to treatment of various forms of cancer.^11–16^ In most cases this has been achieved indirectly through the analysis of localized magnetic resonance spectra of the hyperpolarised agent, the results of which are indicative of the spatial distribution of the molecule, as well as that of its metabolites.^12,16–20^ As many pathological processes, such as ischaemia, cancer or renal disease manifest themselves through changes in tissue pH, this can be reflected in MR spectra through changes in the relative amplitude and chemical shift of the peaks. The use of pH sensitive molecules, in conjunction with MRI and hyperpolarisation, therefore reflects a potentially excellent route to probe different metabolic pathways and aid in disease diagnosis.

This approach has successfully been applied to the study of a broad range of pathologies, using DNP as the magnetisation source^12,21^ and the fast development of pH sensitive agents for PHIP MRI shows that such a strategy can also be applied together with *para*hydrogen induced polarisation.^22^ SABRE hyperpolarisation of pH sensitive molecules has also been reported, with remarkable results being achieved using ^15^N-labelled compounds such as imidazole^23^ or diazirines^24^ as pH probes in conjunction with ^15^N SABRE hyperpolarisation. While no study of the effect of pH on SABRE hyperpolarised ^1^H NMR spectra has yet been published, Moreno and co-workers have observed that pH has a strong influence on the amplitude of solvent signals when the magnetisation transfer catalyst is present in solution.^25^ Furthermore, work by Tessari et *al*. on the quantification of the SABRE response^26^ has resulted in the production of a new tool for analysis which might be augmented by manipulating pH in the way described in this work to further to improve NMR sensitivity and detection.

We describe here the SABRE hyperpolarisation of vitamin B3 (nicotinic acid (**NA**)) and the effects pH plays on polarisation transfer to its ^1^H, ^13^C and ^15^N nuclei by probing appropriate NMR spectra in methanol-*d*_4_. We show that when **NA** is reacted with [IrCl(IMes)(COD)] (**1**) (where IMes = 1,3-bis(2,4,6-trimethylphenyl)imidazole-2-ylidene, and COD is cyclooctadiene) a SABRE active catalyst is successfully formed and we analyse its performance as a function of substrate loading and polarisation transfer field (PTF). Furthermore, we demonstrate that, by altering the pH of the solution, a new polarisation transfer catalyst is formed which has far superior performance to that of the parent. Moreover, the field dependence of the multiple spin terms created under SABRE, as well as the different relaxation and exchange rates that are found when moving from acidic to basic solution as a consequence of the change in catalyst speciation are themselves shown to be diagnostic.

We also demonstrate that the variation in the chemical shift of the **NA** resonances with pH is preserved under SABRE, not only for ^1^H, but also for ^13^C and ^15^N which shows that **NA** has significant potential as a pH probe. We undertake p*K*_a_ assessments based on these changes and conclude by presenting ^1^H-MRI and ^13^C-MRI images of hyperpolarised **NA** in phantoms, which exhibit pH dependent intensity and contrast.

## 2. Experimental

### 2.1 Materials

All of the experimental procedures associated with this work were carried out under nitrogen using standard Schlenk techniques. The solvents used were dried using an Innovative Technology anhydrous solvent system, or distilled from an appropriate drying agent under nitrogen. The catalyst precursor [IrCl(IMes)(COD)] (**1**) employed in this work was synthesized by established procedures.^27^

The SABRE experiments undertaken used a variety of concentrations of the substrate and catalyst that are detailed appropriately in the text. Deuterated methanol (methanol-*d*_4_) was purchased from Sigma Aldrich. Nicotinic acid (**NA**, Sigma Aldrich) and caesium carbonate (Cs_2_CO_3_) (Alfa Aesar) were used as supplied.

### 2.2 Instrumentation and procedures

All the NMR measurements were recorded on Bruker Avance III series 400 MHz or 500 MHz systems. NMR samples were prepared in 5 mm NMR tubes that were fitted with Young's valves. Samples were degassed prior to *p*-H_2_ (3 bars) addition. NMR characterization data was collected using a range of 1-D and 2-D methods that included nOe, COSY and HMQC procedures.^28–32^

### 2.3 SABRE analysis

NMR samples were prepared in 0.6 ml of methanol-*d*_4_. Arrays of NMR measurements were collected using either 20 equivalents of substrate (effective 17-fold excess relative to the catalyst) to 5 mM of iridium (**1**) or 20 equivalents of substrate and 20 equivalents of base to 5 mM iridium (**1**). After adding *p*-H_2_ at a 3 bar pressure, ^1^H NMR spectra were recorded using a π/2 excitation pulse immediately after sample introduction into the high-field magnet which was preceded by the shaking of the sample in a lower field, typically 65 G. Enhancement factors were calculated from the ratio of the integral areas of the individual resonances in the hyperpolarised spectrum and the spectrum collected under normal H_2_ where the signals arise from magnetisation that is created under Boltzmann equilibrium conditions. More detailed information about the sample preparation step, and the procedures used, can be found in Section 1 of the ESI.†

### 2.4 Polarisation transfer field experiments

The dependence of SABRE with polarization transfer field (PTF) was examined by completing a series of measurements in an automated flow system that allows for repeated sample hyperpolarisation after exposure to *p*-H_2_ in the presence of a predefined low field as described by Mewis and coworkers.^10^ These samples contained 5 mM of **1**, and varying amounts of **NA**, up to a 17-fold excess relative to iridium, and Cs_2_CO_3_, in 3 ml of methanol-*d*_4_. After activation, the samples were introduced into the flow system and simple pulse-collect, as well as multiple-quantum filtered NMR experiments^33^ were performed at PTFs that ranged from 0 to 140 G, in steps of 10 G. The effect of the Earth's magnetic field was screened by completing this process in a μ-metal shield.

### 2.5 EXSY measurements and kinetic analysis

A series of exchange spectroscopy (EXSY) measurements were undertaken to probe the dynamic behaviour of these systems.^32^ This process involved the selective excitation of a bound resonance and the subsequent measurement of a ^1^H NMR spectrum at time, *t*, after the initial pulse. The resulting measurements consisted of a series of data arrays such that *t* takes between 10 and 25 values, typically between 0.1 to 1.0 s, to encode the reaction profile. The precise values were varied with temperature to match the speed of the process. Data were collected for a range of temperatures and sample concentrations. Integrals for the interchanging peaks in the associated ^1^H EXSY spectra were then converted into a percentage of the total detected signal and modelled as a function of *t* to extract the rate of change (see Section 1 of the ESI, Fig. S1 and S2†).

## 3. Results and discussions

### 3.1. Zwitterionic forms of nicotinic acid as a function of solvent

**Scheme 1:**
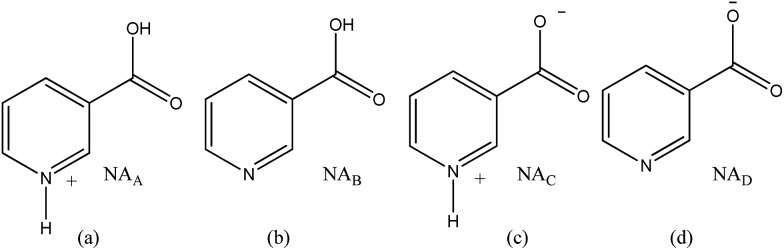
Possible forms of **NA** in solution; (a) conjugate acid (**NA_A_**), (b) uncharged (**NA_B_**), (c) zwitterion (**NA_C_**) and (d) conjugate base (**NA_D_**).

Pyridine carboxylic acids have the capability to undergo multiple protonation events. This leads to the presence of several forms of this molecule in solution (see Scheme 1, neglecting the dication). It is generally assumed that uncharged **NA_B_** reflects a minor contribution with the dominant form being that of the neutral zwitterion **NA_C_** in aqueous media.^34^ In acidic solution the conjugate acid **NA_A_** predominates, whilst in basic environments the conjugate base **NA_D_** is essentially the only form present. These forms co-exist at intermediate acidities.^34^

In the context of SABRE, when **NA** reacts with **1** to form an active polarisation transfer catalyst, it might therefore be expected to exist in one of several forms in solution. This translates into the potential for a highly complex speciation of the resulting magnetisation transfer catalyst, which might also be expected to be affected by the effective p*K*_a_ of the carboxyl function of the bound ligand. The effect of this change is considered here in order to assess its impact on SABRE. Furthermore, given that the bound and free ligands of **NA** are undergoing exchange, the impact of this process on the observed chemical shifts of **NA** is also considered.

Samples containing 100 mM of **NA** were prepared in solutions of neat D_2_O and neat methanol-*d*_4_, as well as the D_2_O/methanol-*d*_4_ mixtures in the proportions 20–80, 40–60, 60–40 and 80–20. We use the well-established technique of pH dependent chemical-shift variations to look at this effect. The corresponding ^1^H NMR chemical shifts of **NA** are summarized in Table 1.

**Table 1:** ^1^H NMR chemical shifts of the indicated protons of **NA** as a function of solvent

	100% D_2_O	80 : 20 D_2_O : MeOD	60 : 40 D_2_O : MeOD	40 : 60 D_2_O : MeOD	20 : 80 D_2_O : MeOD	100% MeOD
H-2	9.13	9.14	9.14	9.13	9.13	9.14
H-4	8.84	8.85	8.78	8.62	8.48	8.43
H-5	8.09	8.07	7.96	7.80	7.66	7.58
H-6	8.91	8.90	8.83	8.80	8.77	8.75

It can be seen from these data that as the amount of D_2_O decreases, in favour of methanol-*d*_4_, the resonances of **NA**, with the exception of H-2, exhibit an upfield shift. The size of this shift is just 0.03 ppm for H-6 but for H-4 and H-5 it increases to 0.05 and 0.06 ppm respectively. These small changes are therefore consistent with **NA_C_** still dominating in methanol-*d*_4_ solution. Interestingly there are far larger shifts in the mixtures where the hydrogen bonding network is clearly affected. We use the expression effective p*K*_a_ throughout this work to reflect the fact that our measurements are done in methanol rather than water.

### 3.2. Reaction of **NA** with **1** in methanol-*d*_4_

It has previously been reported that when **1** reacts with pyridine (py) in methanol-*d*_4_ it forms [Ir(COD)(IMes)(py)]Cl.^9^ When **NA** reacts with **1** in methanol-*d*_4_ the solution rapidly changes colour from yellow to light orange due to the formation of analogous [Ir(COD)(IMes)(**NA_B_**)]Cl (**2a**) where the **NA** ligand binds through nitrogen in its neutral form. When the ratio of iridium to **NA** is 1 : 7 NMR spectra at 245 K reveal that this reaction goes 96% to completion. These NMR data show sharp peaks for the bound, non-exchanging, **NA** resonances of **2a** which has been characterised by ^1^H, ^13^C and ^15^N NMR spectroscopy and the results are detailed in the ESI† (Section 2.12.1). The ^13^C signal for the pyridyl quaternary carbon appears at *δ* 168 and indicates that the carboxyl group is protonated.

### 3.3. Addition of H_2_ to [Ir(COD)(IMes)(**NA_B_**)]Cl (**2a**) in methanol-*d*_4_

**Scheme 2:**
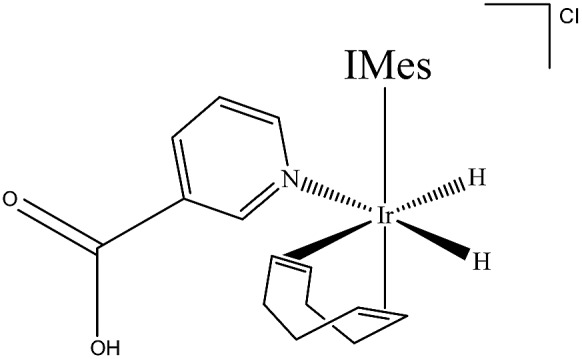
[Ir(H)_2_(COD)(IMes)(**NA_B_**)]Cl (**3a**), major product formed by H_2_ addition to **2a** in methanol-*d*_4_ solution.

A methanol-*d*_4_ solution of **2a**, in which the **NA** ligand binds in its neutral form, was then exposed to a 3 bar pressure of hydrogen gas at 245 K. The ensuing reaction led to detection of two isomers of the H_2_ oxidative addition product [Ir(H)_2_(COD)(IMes)(**NA_B_**)]Cl. The dominant form of this product, **3a**, yields two diagnostic hydride resonances at *δ* –11.91 and *δ* –17.32 (ESI,† Section 2.12.3). These hydride ligands lie *trans* to the N-heterocycle and COD respectively. This conclusion is supported by the corresponding 2D-^1^H COSY NMR spectrum, which contains a cross-peak between the hydride ligand signal at *δ* –17.32 and the bound H-2 and H-6 **NA_B_** resonances at 8.70 and 8.84 respectively, which are *cis* to the hydride. In this case, **3a** forms because addition is favoured over the axis containing the weak nitrogen donor, rather than the strongly donating IMes ligand (Scheme 2).

### 3.4. Formation of [Ir(H)_2_(IMes)(**NA_B_**)_3_]Cl (**4a**) in methanol-*d*_4_ and its SABRE ^1^H NMR signal enhancement activity

**Fig. 1:**
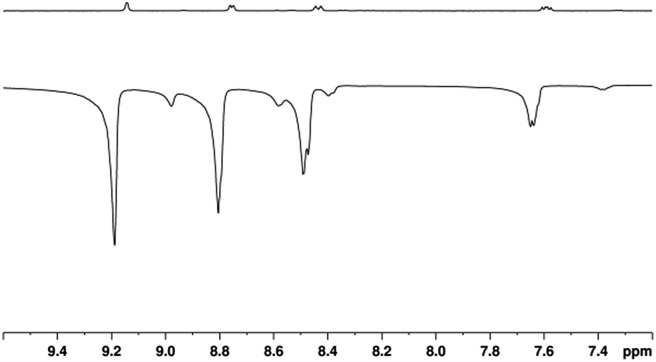
^1^H NMR spectra of a solution containing **4a** prepared with 17-fold excess **NA** that are acquired under Boltzmann equilibrium conditions (top) and after hyperpolarisation *via* 3 bar of *p*-H_2_ (bottom).

**3a** then reacts further with **NA** to form **4a** which is directly analogous to the previously reported SABRE active complex, [Ir(H)_2_(IMes)(py)_3_]Cl.^35^ Characterisation data for **4a** is presented in the ESI,† Section 2.12.5. When **4a** is examined under *p*-H_2_, SABRE is observed in the corresponding high-field NMR spectra. This effect is readily evident when a sample containing 5 mM of **1** and 20 equivalents of **NA** (17-fold excess relative to **4a**) is examined as detailed in Fig. 1.

A series of one-shot ^1^H NMR spectra were then collected on this sample, using π/2 read-out pulses, and substantial signal enhancements were observed for all four of the non-exchangeable resonances of free **NA**, alongside those for its equatorially bound counterparts in **4a**, and its hydride ligands.

The largest ^1^H NMR signal enhancement levels seen for the free substrate resonances with **4a** were observed for H-2 (–52 fold), followed by H-6, H-4 and H-5 (–41, –31 and –21 fold respectively upon observation at 400 MHz). These enhancement levels are always smaller than those of the corresponding bound equatorial **NA_B_** signals of **4a**. For example, in the case of H-2, this difference is reflected in a –92-fold signal gain rather than the –52-fold response. All of these values, and their errors, are presented in Table S1 of the ESI.†

In order to assess the effect that substrate loading has on these SABRE ^1^H NMR enhancements, samples containing 5 mM of **1** and increasing amounts of **NA** were prepared; the amount of substrate added ranging from 4 to 20 equivalents (up to 17-fold excess relative to **4**).

**Fig. 2:**
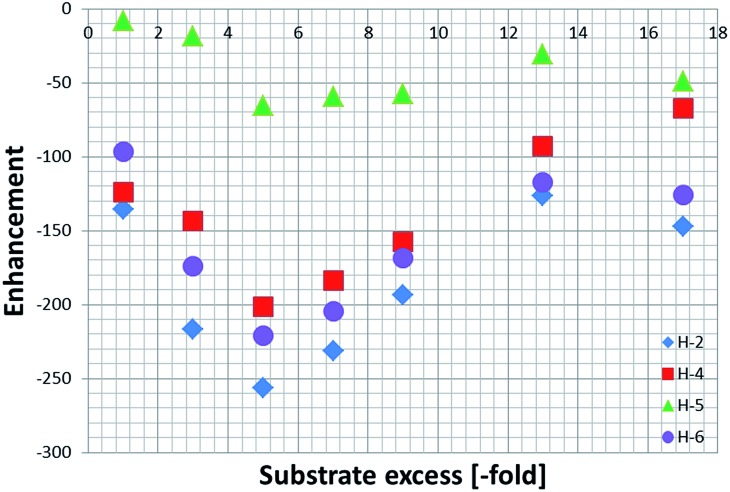
Total ^1^H NMR SABRE signal enhancements achieved by **4a** as a function of the **NA** excess. The indicated values reflect the sum of the signals for the corresponding free and equatorial **NA** resonances of **4a**.

These enhancements were then quantified as detailed in the Experimental section and are summarised in Fig. 2. As expected, the largest signal enhancements are obtained for the *ortho* resonances (H-2 and H-6) of **NA** as they correspond to the proton which is located closest to the binding site in **4a**.^6^

When analysing the effect of the substrate concentration in solution on the enhancement values, we observe a build-up towards the maximum value of –744 ± 35, which is obtained for a substrate loading of 40 mM (5-fold excess), followed by a decay as the ligand excess is further increased. This trend can be explained by analysing the influence of ligand concentration on the exchange rates as detailed shortly.

### 3.5. Bulk **NA** build-up rates for [Ir(H)_2_(COD)(IMes)(**NA_B_**)_3_]Cl (**4a**) as a function of substrate excess in methanol-*d*_4_

In order to rationalise the change in enhancement level with ligand loading, we quantified the free **NA** build-up rate in solution as function of substrate excess. The corresponding values were found to fall from 4.27 s^–1^ to 3.78 s^–1^ across this series (Fig. S4†). This small difference in rate was nonetheless larger than the errors in these values (ESI, Table S2†) and reflects a change in ligand exchange behaviour. The associated acid-base equilibria of **NA** must therefore be affected by this change in loading as the rate for a dissociative process is independent of ligand excess. For example, if more **NA** present in solution were to become protonated *via* the bound **NA_B_** contained within **4a** then the new complexes Ir–N bond strength would increase and a reduction in the free **NA** build-up rate would be seen. This hypothesis can be verified by using NMR pH titration curves to determine the effective p*K*_a_ values for the free and bound environments in this solvent.

In the following sections we present a series of such pH studies that are used to examine the influence of pH on the form of the SABRE magnetisation transfer catalyst, as well as on the associated ligand build-up rates, enhancements and their dependence on the polarisation transfer field. We then link these studies to effective p*K*_a_ determinations through the pH titration method.

### 3.6. Formation of [Ir(COD)(IMes)(**NA_D_**)]Cl (**2b**) in methanol-*d*_4_

When a methanol-*d*_4_ solution of **1** containing an excess of **NA** and Cs_2_CO_3_ is examined by NMR spectroscopy, the formation of [Ir(COD)(IMes)(**NA_D_**)]Cl (**2b**) rather than **2a** is revealed. **2b** was characterised by NMR spectroscopy (ESI,† Section 2.12). This reaction now goes to completion at 245 K, and the ^13^C chemical shift of the pyridyl quaternary carbon becomes *δ* 164 due to its binding as **NA_D_**. This change in speciation can also be replicated by adding Cs_2_CO_3_ to a pre-prepared solution of **2a**.

### 3.7. Addition of H_2_ to [Ir(COD)(IMes)(**NA_D_**)]Cl (**2b**) in methanol-*d*_4_ to form [Ir(H)_2_(IMes)(**NA_D_**)_3_]Cl (**4b**)

A methanol-*d*_4_ solution, where the preformed **2b** to **NA** ratio was 1 : 19, was prepared and then exposed to a 3 bar pressure of hydrogen at 245 K. The reaction of **2b** with H_2_ led to the formation of [Ir(H)_2_(COD)(IMes)(**NA_D_**)]Cl (**3b**) which is directly analogous to **3a**. This complex was identified by ^1^H NMR spectroscopy and yields two coupled, and diagnostic, hydride resonances, at *δ* –12.18 and *δ* –17.37 (ESI,† Section 2.12.4). Analogously to **3a**, the 2D-^1^H COSY NMR spectrum exhibits a cross peak between the hydride resonance at *δ* –17.37, and the bound H-2 and H-6 **NA_D_** resonances at *δ* 8.53 and 8.85 respectively. A single hydride resonance, corresponding to **4b** can be detected upfield, at *δ* –22.36 in this sample (ESI,† Section 2.12.6). The absence of any other hydride peaks indicates that **NA_D_** binds more effectively than **NA_B_**, and that our original hypothesis of a complex metal speciation is not seen in practice. This is confirmed by the trends in enhancement values described as a function of pH in Section 2.4.

### 3.8. Rationalising the coordination of **NA**: effect of pH on the NMR titration curves of the free nicotinic acid in methanol-*d*_4_

In order to determine the effective p*K*_a_ values for the free and bound resonances of **NA** more generally, and to study the effect of pH on the behaviour of nicotinic acid in terms of chemical shift and SABRE enhancement, a set of samples, containing 5 mM of **1**, 100 mM of **NA** and increasing amounts of Cs_2_CO_3_, ranging from 0 to 100 mM, under 3 bars of H_2_ were prepared.

**Fig. 3:**
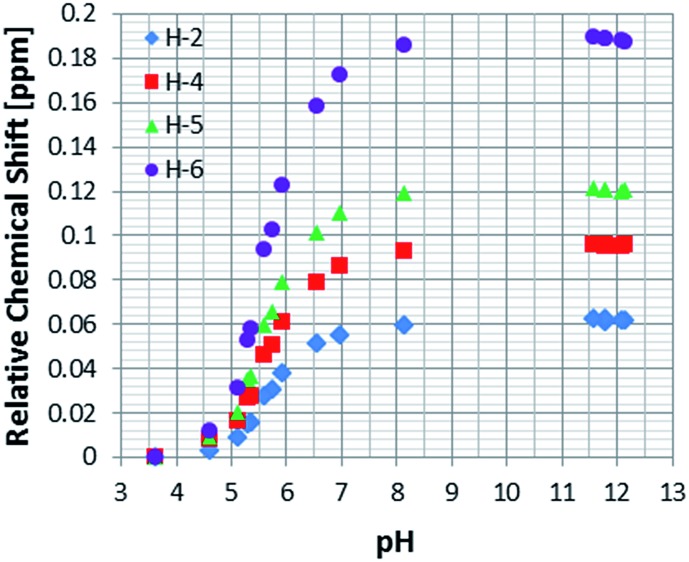
^1^H NMR titration curves measured using the indicated free **NA** resonances in the presence of **4** in methanol-*d*_4_.

This process allowed us to monitor the chemical shift variation (Δ*δ*) for the free and bound resonances of **NA** over the pH range 3.6–12.5 (as measured by a standard pH electrode). These values are presented in Table S4 (see ESI†) and depicted in Fig. 3 for free **NA**. These data show that all of the free **NA** resonances move upfield with increasing pH, the smallest shift being exhibited by H-2 (Δ*δ* = 0.06) and the largest by H-6 (Δ*δ* = 0.18 ppm) in accordance with the likely increase in deshielding due to increased ring current in the anion. Protons H-4 and H-5 exhibit maximum chemical shift differences of 0.09 and 0.12 ppm respectively.

**Fig. 4:**
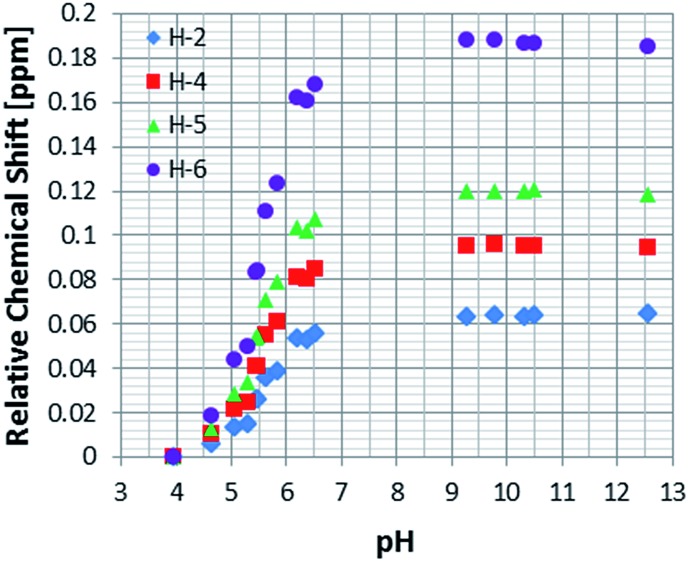
^1^H NMR titration curves measured for the resonances of **NA** in methanol-*d*_4_ solution without catalyst.

As a control, to see if these chemical shift changes were influenced by the presence of the catalyst in these methanol-*d*_4_ solutions, a series of analogous samples were prepared without catalyst (Fig. 4). All the free **NA** resonances again exhibit an upfield shift that is largest for the H-6 site. Upon passing pH 8, a plateau is reached and no further variation with added base is seen. It is possible to conclude that the values obtained for the relative chemical shifts of the free protons in the absence of catalyst are comparable to those seen with it. Hence, this behaviour can be analysed solely on the basis of the relative amounts of the different forms of **NA** illustrated in Scheme 1.

The associated effective p*K*_a_ values were then extracted *via* a Henderson–Hasselbach analysis^36,37^ using the equation:
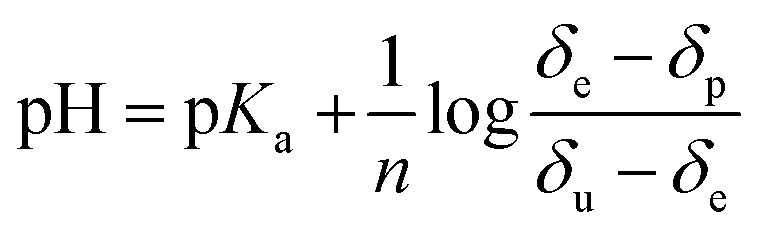
where pH is the value measured using a standard electrode, *δ*_e_ is the chemical shift value determined from the NMR spectra at different pH values, *δ*_p_ is the chemical shift of **NA_C_**, *δ*_u_ is the chemical shift value corresponding to the anion **NA_D_** and *n* is the Hill coefficient.

Data analysis reveals that the effective p*K*_a_ values determined for **NA_C_** in methanol-*d*_4_ in the presence of **4** are 7.06, 6.67, 6.75 and 6.75 for H-2, H-4, H-5 and H-6 respectively. They should all be the same and an error of 0.2 pH units is therefore estimated. A graphical representation of the Henderson–Hasselbach curves for each proton is presented in the ESI (Fig. S10–S12†).

### 3.9. Effect of pH on the NMR titration curves of the bound nicotinic acid resonances in methanol-*d*_4_ in **4**

**Fig. 5:**
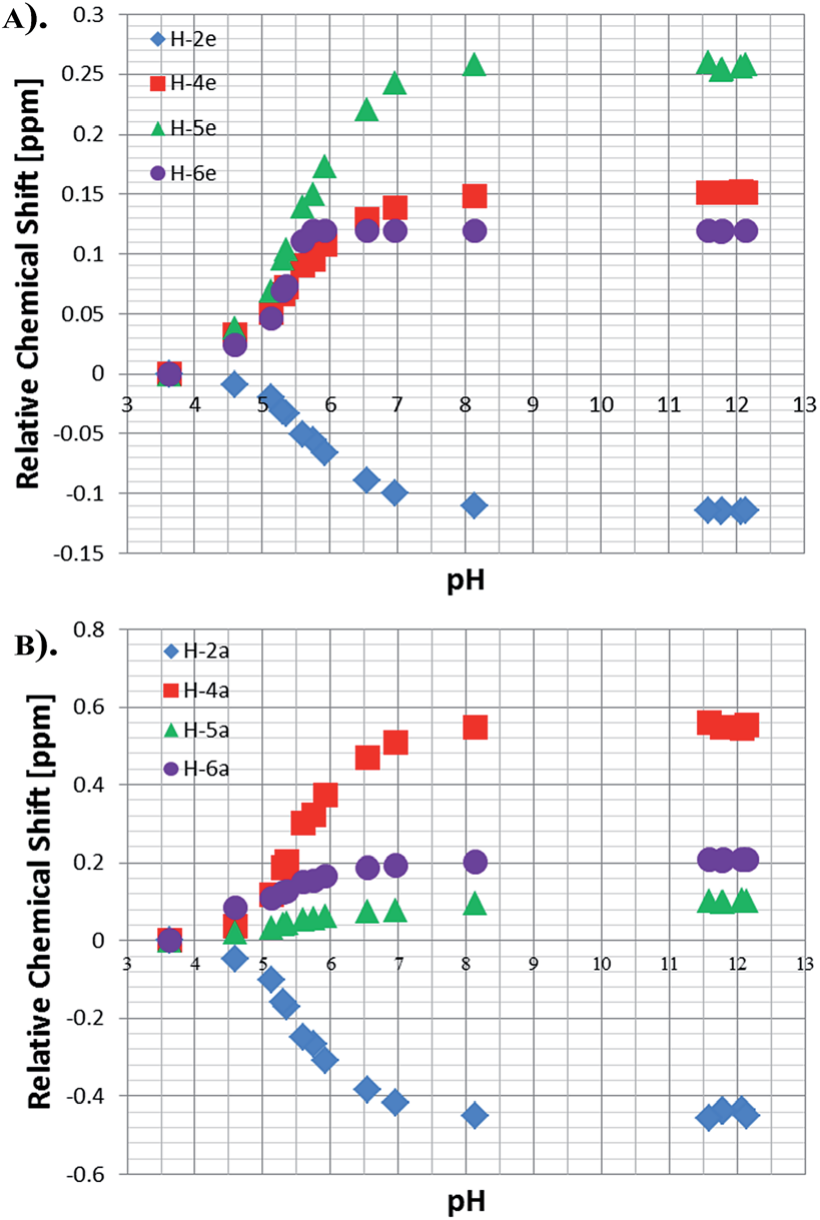
Relative chemical shifts for the bound **NA** resonances of **4**: (a) equatorial protons and (b) axial protons in methanol-*d*_4_.

NMR titration curves were also completed for the equatorially and axially bound resonances of **NA** in **4**. Data analysis shows a significant change in the behaviour of the H-2 resonance which consistently shifts down-field with increase in pH, exhibiting maximum Δ*δ* values of –0.11 and –0.43 for the equatorial and axial site respectively (Fig. 5). The corresponding Δ*δ* values for H-4 are 0.55 and 0.15 ppm, while for H-6 they are 0.20 and 0.12 respectively. These changes compare to those seen for free **NA** of 0.06, 0.18, 0.09 and 0.12 H-2, H-6, H-4 and H-5 respectively.

The effective p*K*_a_ values for bound **NA** in the axial and equatorial sites were estimated in a similar way to that already described for free **NA**. For the axial position, the effective p*K*_a_ values derived from H-2 and H-4 are 6.05 and 6.20 respectively, and contrast with those determined from H-5 and H-6 for which the values are 3.78 and 3.53 respectively. The values obtained from the equatorial **NA** protons were 5.58, 4.57, 5.05 and 6.07 for H-2, H-4, H-5 and H-6 respectively, and are all lower than those of free **NA**. This confirms our previous hypothesis that the reduction in build-up rates seen with high ligand loadings is due to the acidity of the bound **NA** ligands which causes the free **NA** to act as a base. The reason for the high variability in effective p*K*_a_ value is clearly associated with the strength and type of metal bonding interaction in addition to the effect that is introduced by **NA** interacting in either the neutral of conjugate base forms. The absolute chemical shift values determined here therefore reflect a combination of both shielding and ring current effects which are modified through inductive and mesomeric changes.

The larger chemical shift changes are observed in the axial plane where the **NA** is *trans* to the carbene which is a good σ donor and π acceptor. In contrast, the two ligands in the equatorial position are both *trans* to the hydride, which is purely a σ donor and strong *trans*-labializing ligand, the latter change lengthening the Ir–**NA** bond and accounting for its lability.

Another way of probing the strengths of the substrate's interaction with the metal centre is by examining the difference between the free and bound ^15^N chemical shifts of **NA**.^38^ At pH 3.6 these correspond to Δ*δ*_ax_ and Δ*δ*_eq_ values of 60.1 and 42.9 ppm for the axial and equatorial sites respectively. In contrast, the corresponding values at pH 12.5 are 61.0 and 44.1 ppm. This change confirms that formation of the expected carbanion in alkaline solution leads to stronger Ir–N bonding. The ligand build-up rates probe this effect directly, falling with added base as the Ir–N bond increases in strength (see following section and Table S6, Section 2.6 of the ESI†). Furthermore, the ^13^C chemical shifts of the bound **NA**'s aromatic CH resonances also move with pH by 1–2 ppm units to higher field on forming the anion (as detailed in Section 2.12.5 and 2.12.6 of the ESI†). These data therefore confirm the proposed change in formulation of **4a** and **4b** with pH.

### 3.10. Effect of pH on the free-ligand build-up rate and activation parameters of [Ir(H)_2_(IMes)(**NA_B_**)_3_]Cl (**4a**) and [Ir(H)_2_(IMes)(**NA_D_**)_3_]Cl (**4b**) in methanol-*d*_4_

The rates of ligand transfer from the key equatorial site into solution have been measured for **4a** and **4b** as a function of temperature. These results are presented in detail in Table S6 (see ESI†). The associated rate constant for **4a** proved to be 6.98 ± 0.07 s^–1^ at 300 K whilst for **4b** it was 3.33 ± 0.01 s^–1^ at the same temperature. The corresponding activation parameters for these free **NA** build-up rates in solution are presented in Table 2. These data suggest that the binding energy of **NA** in both **4a** and **4b** is actually lower than that of pyridine in the analogous complex [Ir(H)_2_(IMes)(pyridine)_3_]Cl, for which the site *trans* to hydride has a Δ*H*≠(build-up) value of 95 ± 1 kJ mol^–1^.^35^

**Table 2:** Activation parameters for free **NA** build-up in methanol-*d*_4_ solution

Activation parameters	**4a**	**4b**
Δ*H* ^≠^ (kJ mol^–1^)	88	88
±	2	3
Δ*S* ^≠^ (J K^–1^ mol^–1^)	70	64
±	8	12
Δ*G*≠300 (kJ mol^–1^)	66.9	69.4
±	0.4	0.3
*R* square	0.998	0.998

Given that the average p*K*_a_ of pyridine is 5.17 and that of **NA** is reported to be 5.00,^34^ we attribute the lower build-up rates to steric effects that act to lengthen the **NA** bond in the ground state and thereby reduce the subsequent entropy gain of ligand loss and the associated solvation changes.^39^ This effect is felt most strongly for highly solvated **4b**. Analogous ligand build-up rates were also measured for samples containing 5 mM of **4b**, prepared with increasing amounts of **NA** and Cs_2_CO_3_ in equal proportion (Fig. S15, Section 2.5 of the ESI†). The corresponding rates increase from 0.93 ± 0.01 s^–1^ (obtained for the sample prepared with 1-fold excess of substrate and 1-fold excess of base respectively) to 1.78 ± 0.11 s^–1^ (17-fold excess of substrate and 17-fold excess of base) as the expected plateau is reached for this dissociative process. The rate of free-**NA** build-up is therefore 1.78 ± 0.11 and smaller than that in an analogous sample without base (4.27 s^–1^). This situation reflects the greater stability of the complex when **NA** binds as the anion **NA_D_**. The initial under assessment of the exchange rate results from the fact that the EXSY method misses an exchange event if a molecule of the originally excited **NA**_bound_ returns to the complex rather than remaining in bulk solution. As the substrate excess increases, the chance of this degenerative process happening reduces.

### 3.11. pH influence on proton relaxation times in methanol-*d*_4_

In order to assess the effect pH manipulation has on the signal lifetimes of the ligand, we have also examined the effect of pH on the relaxation times of the free **NA** protons at pH 3.6 and pH 12.5.

The corresponding *T*_1_ values are listed in Table 3. It can be seen that the isolated proton, H-2, has the longest *T*_1_, with a value of 18.4 s in degassed solution. At pH 12.5, this *T*_1_ value changes to 23.7 s, with the corresponding increase suggesting that the tautomer with a protonated nitrogen plays a role in relaxation at low pH. The presence of **4a** in solution reduces the effective *T*_1_ of H-2 to 10.3 seconds in accordance with its role in SABRE, and the increase to 17.6 at high pH reflects the loss of contribution from the pyridinium tautomer and slower catalyst exchange.

**Table 3:** *T*_1_ values (in seconds) for **NA** measured before and after H_2_ addition, in the absence of O_2_, in methanol-*d*_4_ solution. The errors are below 1%

	H-2	H-4	H-5	H-6
**NA**	18.4	9.6	6.5	11.3
**NA** with Cs_2_CO_3_	23.7	6.5	5.2	8.7
**NA** and **4a**	10.3	8.3	4.9	7.3
**NA** and **4b**	17.6	7.2	4.2	6.0

In addition to these changes, the H-4, H-5 and H-6 sites *T*_1_ values fall by 32, 20 and 23% respectively in the presence of Cs_2_CO_3_ alone. In the presence of the activated complex, at pH 3.6, these values differ from their reference point by 14, 25 and 35% respectively, and they fall further by 25, 35 and 47% at pH 12.5. These observations demonstrate therefore that the most pH sensitive resonances are also the most sensitive to relaxation, while confirming that the catalyst changes the relaxation rates of free **NA** under exchange.

### 3.12. pH influence on SABRE enhancements by **4** in methanol-*d*_4_

When a sample of **1** in the presence of 20 equivalents of **NA** is probed for SABRE at 298 K and a pH of 3.6 (after transfer at 45 G) its four protons exhibit a total Zeeman type (*I*_z_) signal enhancement of 110.

**Fig. 6:**
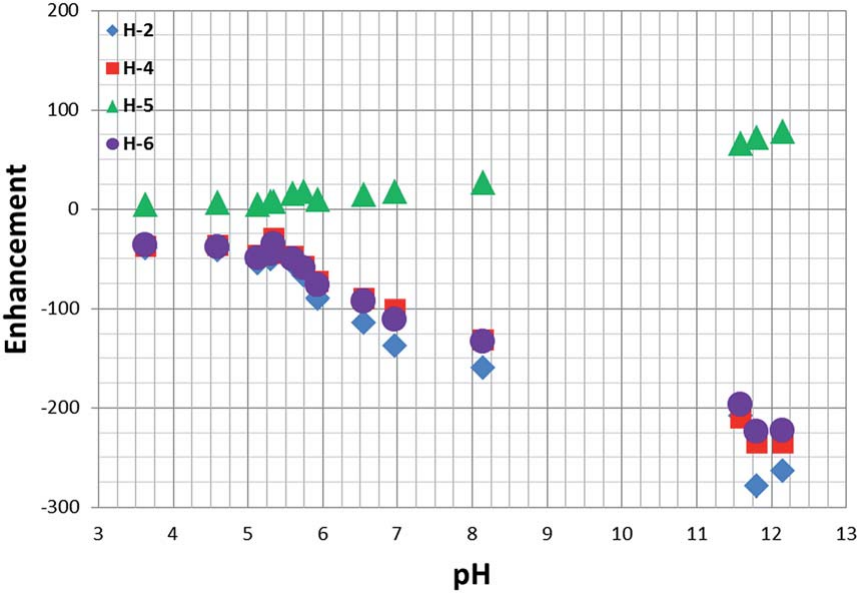
^1^H NMR signal enhancements observed for the free **NA** protons as a function of pH.

However, upon moving from pH 3.6 to pH 8 the observed signal enhancement increases to *ca.* 400 *via* a sigmoidal variation in signal gain with pH in a similar way to that described for the chemical shift profiles (Fig. 6).

When the pH is increased to 12, the total **NA** signal enhancement increases to over 800-fold. This change is likely to result from the formation of methoxide through an iridium promoted reaction with caesium carbonate and there is therefore the potential to change the active catalyst again. It should be noted that the linewidth of the H-2 resonance for free **NA** narrows as the pH changes from 3 to 12 in accordance with the observed increase in *T*_1_ value and reduction in contribution from **NA_C_** with a protonated nitrogen.

### 3.13. Probing the effect of the polarisation transfer field on the efficiency of SABRE for transfer to ^1^H, ^13^C and ^15^N in methanol-*d*_4_

As mentioned previously in the introduction, the value of the polarisation transfer field (PTF) can affect the levels of SABRE enhancement that result.^6^ We therefore explored this behaviour through a series of single shot measurement with **4a** and **4b** respectively and describe these results in Sections 2.7–2.11 of the ESI.† **4a** and **4b** proved to behave in a similar fashion, with the maximum ^1^H NMR enhancement level being obtained at around 70 G for H-2 in each case. The enhancement ratios delivered by **4b**/**4a** at 70 G are 5.3, 4.5, 1.9 and 5.5 for H-2, H-4, H-5 and H-6 respectively, and confirm the superior detection levels in basic solution. Similar signal gains have been detected when performing multiple quantum filtered experiments using the only parahydrogen spectroscopy^33^ (OPSY) protocol (see ESI, Section 2.7, Fig. S17–S20†). This gradient-based selection procedure probes the magnetization that is created through SABRE *via* the creation of appropriate zero quantum, single quantum, double quantum, triple quantum and quadruple quantum terms. In this case, the observations confirm that whilst the creation of single spin polarization is most efficient, two, three and four-spin longitudinal spin-order is created and readily detectable.

Additionally, the corresponding ^15^N SABRE response of **4** was detectable without labelling, and the addition of base found to significantly improve it such that **4b** is readily detectable after transfer *via* a PTF of 0.2 G (see ESI,† Section 2.10).

The absolute enhancement for the ^1^H decoupled ^15^N resonance of **4b**, calculated *via* a ^15^N labelled pyridine standard, was 9218-fold. In the corresponding ^13^C spectrum, no enhancement could be detected when analysing transfer *via* **4a**, but the addition of base facilitates the detection of all of the **NA** ring ^13^C resonances *via* **4b**. These results are described in the ESI† (Sections 2.8 and 2.9) and serve to further illustrate the effect of pH manipulation on the hyperpolarised NMR signal intensity.

### 3.14. pH influence on solvent polarisation

Several reports of methanol participating in the active polarisation transfer catalyst can be found in the literature^35,40^ and, as observed by Moreno and co-workers, enhancement of the OH proton can be easily attained in mild acidic conditions.^25^ The residual OH resonance of this solvent is considerably enhanced in the presence of **4a** and its enhancement is shown to decrease with increasing substrate amount from a maximum of approximately 100-fold for up to a 7-fold excess of **NA** to 68-fold for a 17-fold excess of **NA** (ESI, Section 2.11 and Fig. S29†). This fall matches the findings of Lloyd,^35^ Moreno,^25^ and Eshuis^26^ and the observation of these effects serves to further illustrate that alcohols can be polarised by SABRE.

### 3.15. pH influence on the quality of ^1^H and ^13^C derived images

The pH sensitivity of **NA** in the presence of **4a** or **4b** was tested by performing Magnetic Resonance Spectroscopy experiments (MRS) which consisted of acquiring localised spectra of the hyperpolarised agent dissolved in acidic or basic solutions in a voxel of 3 × 3 × 10 mm localised in the centre of the glass phantom used for the experiments, an approach that is analogous to state-of-the-art DNP experiments.^12^ These preliminary results, presented in Section 3.1 of the ESI (Fig. S35†), show that the pH sensitivity of **NA** can be exploited both during the polarisation transfer step (to increase sensitivity by enhancing intensity) and during the detection step (due to its inherent qualities as a pH probe).

**Fig. 7:**
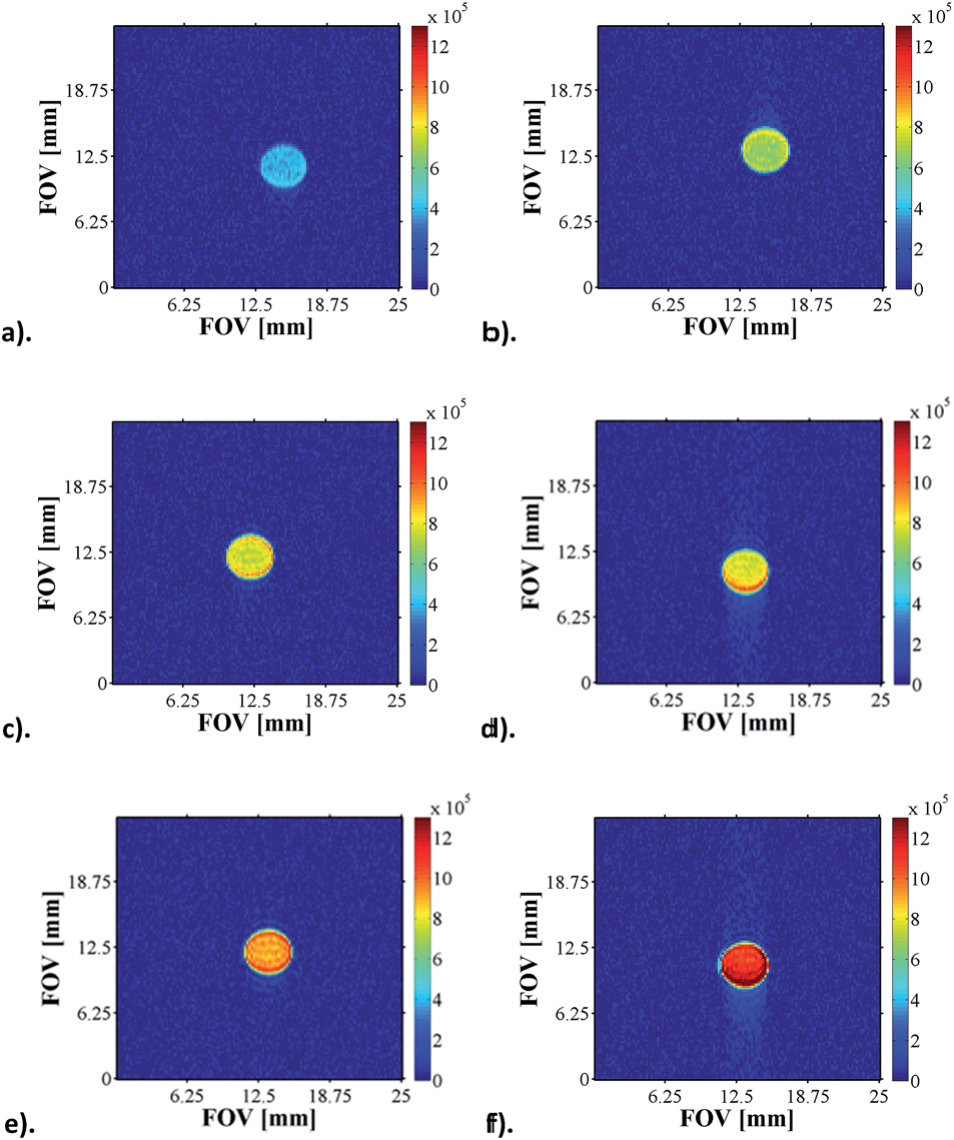
^1^H MRI images of hyperpolarised **NA** as a function of pH: (a) pH 3.6, (b) pH 4.6, (c) pH 5.1, (d) pH 5.7, (e) pH 5.9 and (f) pH 6.9.

Furthermore, we have recorded a series of SABRE images on samples containing 5 mM of **1**, 20 equivalents of **NA** (17-fold excess, relative to **4**) and increasing amounts of base (up to 17-fold excess base). As expected, as the amount of Cs_2_CO_3_ increases, the pH of solution increases and the signal intensity seen in the response increases by one order of magnitude for the same concentration of substrate (Fig. 7).

**Fig. 8:**
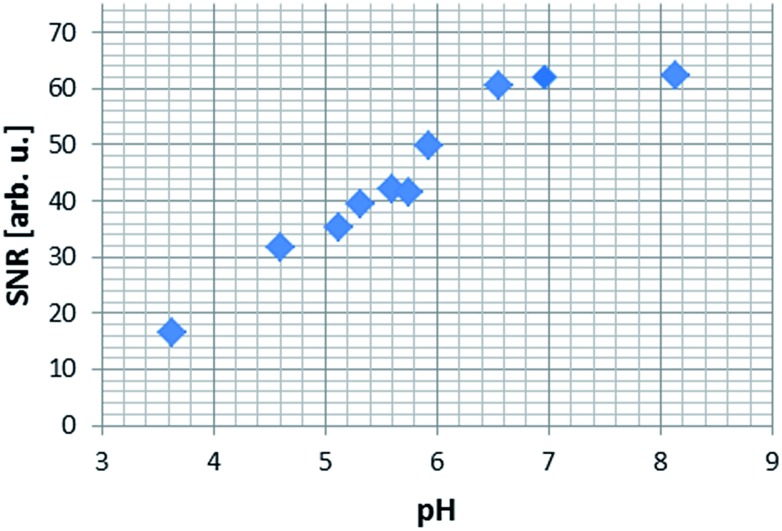
Average signal to noise (SNR) ratio produced in a series of ^1^H MRI images of hyperpolarised **NA** in methanol-*d*_4_ solution.

In order to more precisely quantify this improvement in terms of the image quality, five one-shot images of the hyperpolarised samples were acquired and the average image signal to noise ratios (SNR's) were calculated. The SNR values exhibit an increasing trend up to the point where the pH approaches physiological values, which opens up the possibility of using **NA**, a naturally biocompatible molecule, as an MRI contrast agent for *in vivo* investigations (Fig. 8). As the basicity of the solution is further increased, the corresponding SNR values decrease rapidly, a trend which we attribute to possible transverse relaxation rate effects caused by the methoxide ion.

**Fig. 9:**
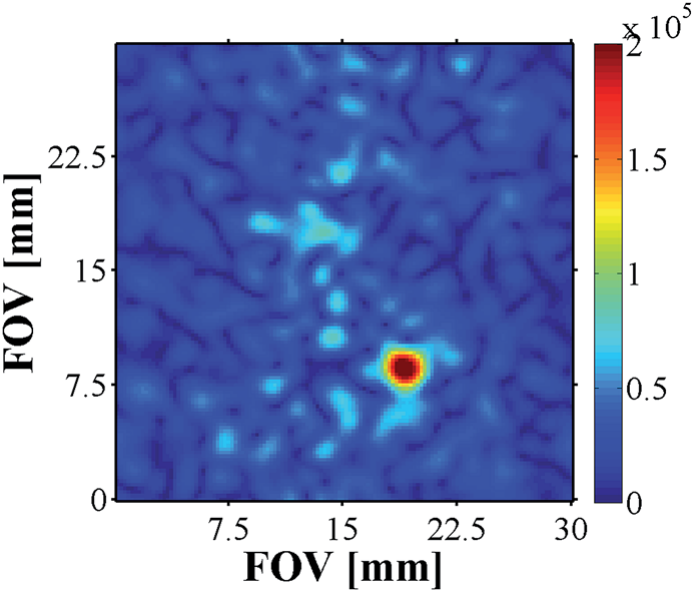
^13^C image of hyperpolarised ^13^C-NA produced by **4b**. Matrix size 64 × 64, slice thickness 10 mm, nominal resolution 470 μm^2^.

^13^C MRI As ^13^C imaging can be used to successfully analyse metabolic processes, as demonstrated by DNP,^14^ we also examined carbonyl-^13^C labelled **NA** with **4a** and **4b** derived samples (see ESI†). The samples contained a 17-fold excess ^13^C-**NA** relative to **4**, and were first hyperpolarised using the automated system described earlier at 0 G prior to the recording of the corresponding ^13^C image using a rapid acquisition scheme. While the images acquired for **4a** did not yield signals above the background noise level, the addition of base, and use of **4b**, facilitated the successful recording of an image (Fig. 9).

## Conclusions

We have established here that nicotinic acid (**NA**) reacts with [IrCl(IMes)(COD)] (**1**) and H_2_ to successfully form a SABRE active catalyst. In acid solution, the carboxyl function of **NA** is protonated when bound to iridium and relatively weak SABRE is observed in the corresponding free **NA** ^1^H NMR signals. In contrast, when Cs_2_CO_3_ is added, to make the resulting solution basic, **NA** binds as its conjugate base with the resulting charge delocalisation acting to stabilise the catalyst. In this case the catalysts longer lifetime leads to dramatically improved SABRE activity.

When analysing the performance of the SABRE catalyst as a function of substrate loading and polarisation transfer field, we have found that the maximum enhancement is obtained using 5-fold excess of ligand at 65 G. In a ^1^H NMR spectrum recorded at 400 MHz the resulting 744-fold signal gain reflects a compression of the total data acquisition time required under normal conditions for an equivalent SNR of over 500 000. We note, however, that it is easier to work at 45 G where all of the signals possess a balanced phase.

A subsequent increase in ligand loading leads to a decrease in enhancement, a phenomenon which we attribute to a change in the **NA** acid–base equilibrium position in methanol solution. We verified this hypothesis by using NMR pH titration mapping, in which a series of measurements were undertaken where increasing amounts of base were added to a constant amount of **NA**, H_2_ and **1**.

By doing so, the pH of the solution changes such that progressive deprotonation of the nitrogen and oxygen centres of **NA** occurs, this leads to a higher probability that **NA** can bind to the catalyst. This change in catalyst formulation has been viewed by mapping the effect on the ligands ^1^H NMR chemical shifts as a function of pH. A series of well-defined pH titration curves were produced from which effective p*K*_a_ values can be estimated for methanol-*d*_4_ solution. The hyperpolarised signals for free **NA** were also used to access its p*K*_a_ under the same conditions. Hence these results reflect an important observation that hyperpolarised ^1^H NMR signals can be used for pH assessment in conjunction with SABRE.

When examining the ligand build-up rates for two extreme pH situations (the catalyst in the absence of base and the catalyst formed with an excess of base), we confirmed that the increase in pH slows down the process of ligand build-up in solution. The lower ligand build-up rates, together with significant changes in the relaxation times, lead to the far superior performance of the polarisation transfer catalyst with excess of base, compared to that of its protonated parent. This effect is readily demonstrated in the enhancement *vs.* pH data of Fig. 6, which shows a considerable increase in polarisation at high pH, as well as in the multiple-quantum experiments that were performed at different PTF values and are described in the ESI.† These data therefore illustrate how the detection limit can be improved by harnessing pH if an analytical study is being completed. By extending this approach through the OPSY protocol, such measurements could be made in protio solvents without the need for solvent suppression. We note that in acid solution, the residual protons of the methanol-*d*_4_ solvent also show substantial polarisation (see ESI† Section 2.11).

Furthermore, the increase in pH has allowed for natural abundance ^13^C and ^15^N spectra to be acquired and, as in the case of ^1^H, larger enhancements were obtained for the basic solution in comparison to its acidic parent. We present ^1^H MRI and ^13^C MRI images of SABRE hyperpolarised **NA** in phantoms that exhibit controlled pH dependent intensity and contrast. In the ESI† we demonstrate that Magnetic Resonance Spectroscopy (MRS) can be used on these phantoms to track the pH change in a voxel of interest. These results are, to the best of our knowledge, the first pH-dependent ^1^H MRI data obtained using SABRE.

This study therefore establishes that when the pH sensitivity of **NA** is combined with the increase in signal gain provided for by SABRE hyperpolarisation, a versatile pH MRI probe will result which is directly analogous to that already demonstrated *in vivo* for DNP-MRI. It also reveals the importance of considering the effect pH plays on catalysis during SABRE.
