# Supplementary material for: SABRE hyperpolarisation of vitamin B3 as a function of pH
Source: Chem Sci. 2016 Dec 7;8(3):2257–66. doi: 10.1039/c6sc04043h (PMC5409243; doi:10.1039/c6sc04043h)
Supplement: Supplementary file 1 [file SC-008-C6SC04043H-s001.pdf]

Electronic Supplementary Material (ESI) for Chemical Science.
This journal is © The Royal Society of Chemistry 2016

SABRE hyperpolarisation of vitamin B3 as a function of pH

Alexandra M. Olaru,[a] Michael J. Burns,[a] Gary G. R. Green[b] and Simon B. Duckett*[a]

1. Experimental

2. NMR Experimental Results

- 2.1 Substrate loading effect on SABRE signal enhancement for NA in the presence of 4a.
- 2.2 Substrate loading effect on ligand build-up rates in the presence of 4a.
- 2.3 pH influence on chemical shift.
- 2.4 pH influence on SABRE signal enhancement.
- 2.5 Base loading effect on ligand build-up rates in the presence of 4b.
- 2.6 Ligand build-up rates in the presence of 4a and 4b as a function of temperature.
- 2.7 Effect of PTF on ^{1}H SABRE signal enhancement.
- 2.8 Effect of PTF on the level of ^{13}C SABRE signal enhancement of nicotinic acid.
- 2.9 Effect of PTF on the level of ^{13}C SABRE signal enhancement of ^{13}C labelled nicotinic acid.
- 2.10 Effect of PTF on the level of ^{15}N SABRE signal enhancement.
- 2.11 pH influence on solvent polarisation.
- 2.12 Complex characterisation by NMR.

3. MRI Experimental Procedures and Results

- 3.1 ^{1}H MRI
- 3.2 ^{13}C MRI

4. References

1. Experimental

Materials All of the experimental procedures associated with this work were carried out under nitrogen using standard Schlenk techniques. The solvents used were dried using an Innovative Technology anhydrous solvent system, or distilled from an appropriate drying agent under nitrogen. The catalyst precursor ([Ir(IMes)(COD)Cl] employed in this work was synthesized by established procedures according to literature methods.[1]

The SABRE experiments used different concentrations of the substrate and catalyst as detailed below. Deuterated methanol (methanol-d_4) was used as the solvent in all cases (Sigma Aldrich). Nicotinic acid (Sigma Aldrich) and Cs_2CO_3 (Alfa Aesar) were used as supplied. ^{13}C labelled nicotinic acid was synthesised as described below.

^{13}C-Nicotinic acid (3-pyridine-^{13}C-carboxylic acid)

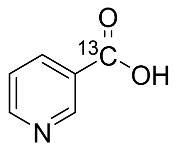

Scheme 1. ^{13}C labelled nicotinic acid.

To a 500 ml 3-neck round bottom flask, fitted with a mechanical stirrer, thermometer, nitrogen line and dropping funnel, was added toluene (12 ml) and *n*-BuLi (2.5M in hexanes, 4.4 ml, 11 mmol) and the solution cooled to –60 °C. A solution of 3-bromopyridine (15.8 g, 10 mmol, 1.27 eq.) in toluene (4 ml) was subsequently added dropwise over 30 minutes, maintaining an internal temperature below –50 °C, resulting in a yellow precipitate. Maintaining the temperature below –50 °C, THF (4 ml) was added, resulting in an orange/brown solution, followed immediately by the addition ^{13}C-DMF (580 mg, 7.83 mmol, 1 eq.). The solution was allowed to warm to ambient temperature over 2 hours and the reaction quenched by the addition of sat. NH_4Cl (10 ml). The layers were separated and the aqueous layer extracted with EtOAc (3 × 10 ml). The combined organic extracts were dried over $MgSO_4$, filtered, and concentrated *in vacuo* to afford crude nicotinaldehyde as orange oil.

A solution of the crude nicotinaldehyde in formic acid (1.61 g, 35 mmol, 5 eq.) was cooled to 0 °C and H_2O_2 (30% in H_2O, 2.4 g, 21 mmol, 3 eq.) added dropwise. The solution was stored at 4 °C overnight then concentrated to dryness *in vacuo* to afford a yellow powder. Vacuum filtration, washing with DCM and ice cold MeOH afforded ^{13}C-Nicotinic acid (908 mg, 93.6%) as a white powder.

^{13}C-NMR (101 MHz, d_6-DMSO): 166.3 (^{13}C), 153.2, 150.2 (d, J = 3.4 Hz), 136.9 (d, J = 2.1 Hz), 126.8 (d, J = 65.4 Hz), 123.8 (d, J = 3.5 Hz); **MS** (ESI) *m/z* 125 [$(M + H)^+$ 100); **HRMS** (ESI) *m/z* $[M + H]^+$ calculated for $^{13}C^{12}C_5H_6NO_2$: 125.0427, found: 125.0428.

Instrumentation and procedures All NMR measurements were recorded on Bruker Avance III series 400 MHz or 500 MHz systems. NMR samples were prepared in 5 mm NMR tubes fitted with Young's valves. Samples were degassed prior to p-H_2 (3 bars) addition. NMR characterization data was collected using a range of 1-D and 2-D methods that included nOe, COSY and HMQC procedures.[2-5]

SABRE analysis NMR samples were prepared containing **4a** or **4b** in 0.6 ml of methanol-d_4. Arrays of NMR measurements were collected using either 20 equivalents of substrate (17-fold excess) to 5 mM of iridium (**4a**) or 20 equivalents of substrate and 20 equivalents of base (17-fold excess) to 5 mM iridium (**4b**). After adding p-H_2 at 3 bar pressure, ^{1}H NMR spectra were recorded using π/2 excitation pulses immediately after shaking the sample in a magnetic field of 45 or 65 G. Enhancement factors were calculated by using the ratio of the integral areas of individual resonances in the hyperpolarised spectrum and the spectrum collected under normal H_2 and Boltzmann equilibrium conditions respectively.

Typical samples reflect the following situations:

Sample 1. 3.1 µM IMes catalyst + 0.25 M substrate (making it a 17-fold excess of **NA** relative to product **4**) in 0.6 ml solvent.

Sample 2. 3.1 µM IMes catalyst + 0.25 M substrate (making it a 17-fold excess of **NA** relative to product **4**) + 0.25 M base (making it a 17-fold excess of Cs_2CO_3) in 0.6 ml solvent.

Field dependence Polarization transfer field (PTF) measurements were recorded using an automated system that allows for repeated hyperpolarisation in the presence of constant low fields of accurate values.[6] Samples containing 5 mM of **4a** and **4b** were prepared in 3 ml of methanol-d_4. After dissolution, the samples were introduced in the flow system and pulse-and-collect, as well as multiple-quantum filtered experiments[7] were performed at PTFs that ranged from 0 to 140 G, in steps of 10 G. The external magnetic field was screened by placing the mixing chamber containing the sample and the PTF coil in a mu-metal shield. Details of the polariser have been reported elsewhere.[6]

EXSY measurements and kinetic analysis A series of exchange spectroscopy (EXSY) measurements were made to probe the dynamic behaviour of these systems.[8] This process involved the selective excitation of a single resonance and the subsequent measurement of a ^{1}H NMR spectrum at time, t, after the initial pulse. The resulting measurements consisted of a series of data arrays such that t is varied between 10 and 25 values, typically between 0.1 to 1.0 s, to encode the reaction profile. The precise values were varied with temperature to suit the speed of the process. Data was collected for a range of temperatures and sample concentrations. Integrals for the interchanging peaks in the associated ^{1}H EXSY spectra were obtained and converted into a percentage of the total detected signal.

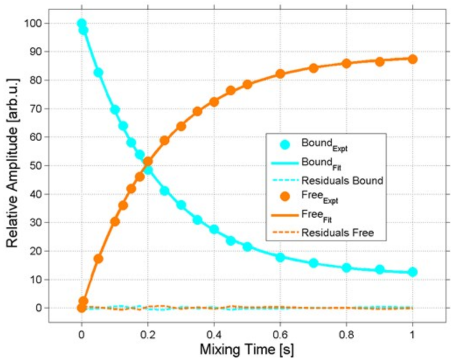

Figure S1. Build-up and decay curves for the ligand loss process for 4a at 295 K.

These data were then analysed as a function of the mixing time according to a differential kinetic model.[9] Rates of exchange were determined by employing a Runge-Kutta[10-11] scheme to solve the system of differential equations and a Levenberg–Marquardt algorithm[12-13] to minimize the sum of the residuals in the associated least mean squares analysis. The theoretical model used to fit the experimental EXSY data involved a two-site exchange (A ↔ B), as expressed by the equations below:

$$-\frac{dA}{dt} = -K_{ab} * A + K_{ba} * B$$

$$-\frac{dB}{dt} = +K_{ab} * A - K_{ba} * B$$

Thermodynamic parameters were calculated using the exchange rates obtained for a wide range of temperatures and the Eyring-Polanyi equation.[14-15]

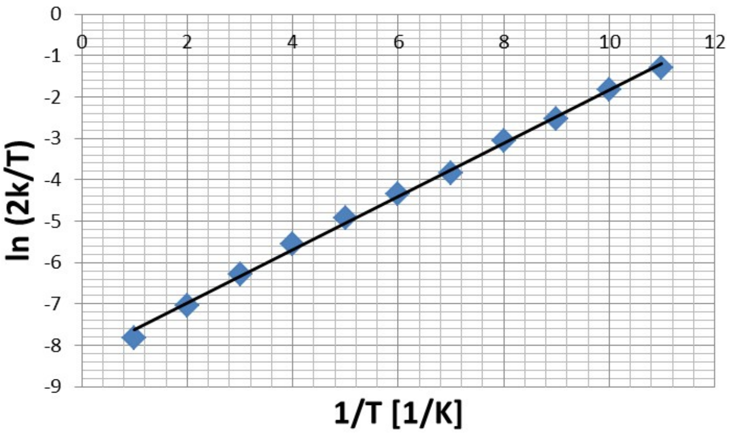

Figure S2. Eyring-Polaniy plot for 4a.

2. NMR Experimental Results

2.1 Substrate loading effect on SABRE signal enhancement for NA in the presence of 4a.

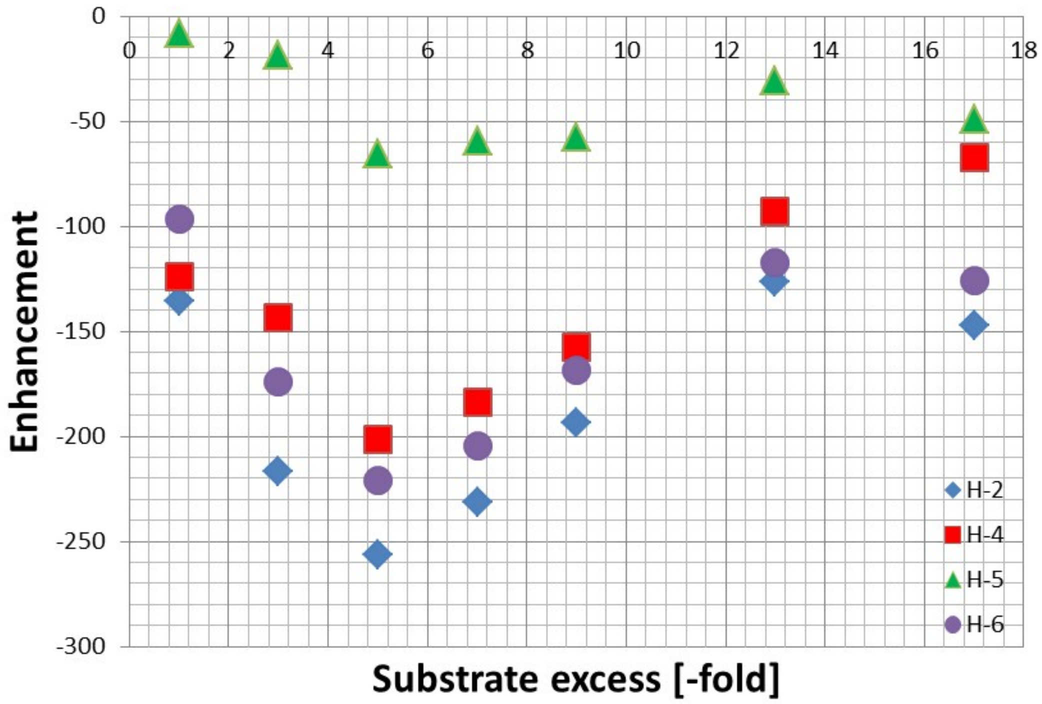

Figure S3. **^{1}H NMR signal enhancements for the indicated protons (free + bound equatorial) as a function of substrate excess.**

Table S1. **^{1}H NMR signal enhancements for the indicated protons as a function of substrate excess.**

Substrate Excess [-fold]	H-2 free	H-2 bound	H-4 free	H-4 bound	H-5 free	H-5 bound	H-6 free	H-6 bound
**1**	-67±11	-69±9	-61±9	-64±4	-6±9	-3±7	-47±5	-51±9
**3**	-109±13	-108±13	-78±8	-66±8	-12±4	-8±3	-70±8	-105±12
**5**	-131±18	-127±18	-107±14	-95±15	-37±8	-29±8	-115±7	-107±16
**7**	-122±10	-110±9	-98±9	-86±7	-35±1	-26±2	-112±9	-94±8
**9**	-95±7	-99±7	-77±5	-81±9	-32±2	-27±4	-88±6	-82±5
**13**	-56±17	-72±21	-45±12	-49±12	-18±8	-14±6	-50±18	-68±28
**17**	-56±21	-92±34	-31±9	-38±20	-21±7	-28±27	-41±14	-86±29

2.2 Substrate loading effect on ligand build-up rates in the presence of 4a.

Table S2. Rates of ligand build-up as a function of NA excess.

Substrate excess (-fold)	k_{4a} [1/s] A→B	k_{4a} [1/s] B→A
**1**	4.27 ± 0.02	4.15 ± 0.02
**3**	4.27 ± 0.01	3.30 ± 0.01
**5**	3.99 ± 0.01	1.33 ± 0.01
**7**	3.87 ± 0.01	0.92 ± 0.01
**9**	3.84 ± 0.01	0.73 ± 0.01
**13**	3.74 ± 0.01	0.46 ± 0.01
**17**	3.79 ± 0.01	0.48 ± 0.01

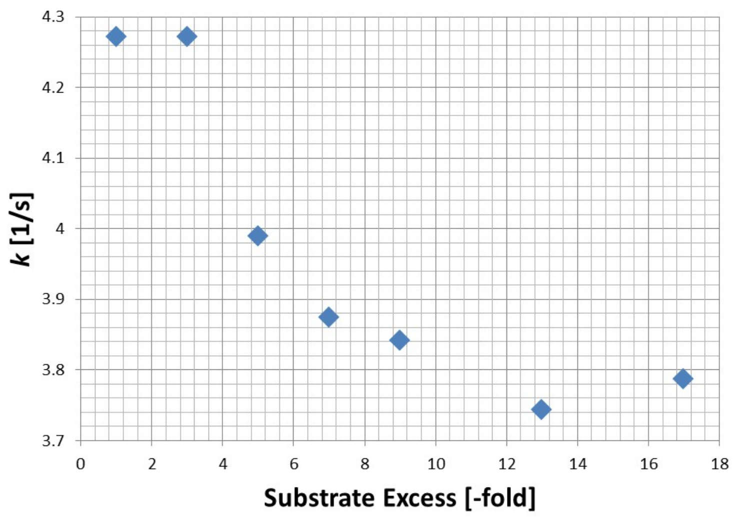

Figure S4. Ligand build-up rates for 4a as a function of NA excess.

The trend associated with the reduction in rate as a function of substrate excess is therefore clear. The errors of Table S2 reflect those associated with the rate analysis and do not include any systematic errors in concentration or potential T_1 effects.

2.3 pH influence on chemical shifts.

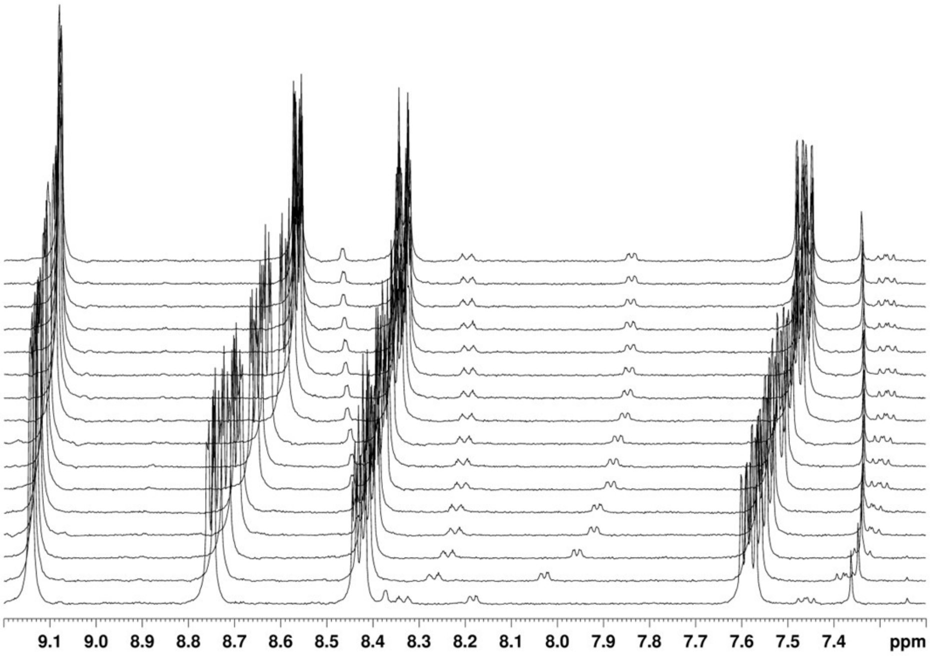

Figure S5. Series of ^{1}H NMR spectra detailing the variation in the free and bound NA chemical shift in 4 as a function of pH.

Table S3. ^{1}H NMR chemical shifts of free NA in the absence of the catalyst as a function of pH.

pH	H-2	H-4	H-5	H-6
3.95	9.144	8.433	7.589	8.755
4.63	9.139	8.423	7.576	8.736
5.06	9.131	8.412	7.560	8.711
5.31	9.129	8.409	7.555	8.705
5.46	9.118	8.392	7.535	8.671
5.47	9.118	8.392	7.534	8.671
5.62	9.109	8.378	7.518	8.644
5.85	9.106	8.372	7.510	8.631
6.21	9.091	8.352	7.485	8.592
6.37	9.092	8.353	7.486	8.594
6.51	9.089	8.349	7.481	8.586
9.29	9.081	8.338	7.469	8.566
9.78	9.081	8.338	7.469	8.567
10.33	9.081	8.338	7.469	8.568
10.51	9.080	8.338	7.468	8.568
12.56	9.080	8.338	7.470	8.569

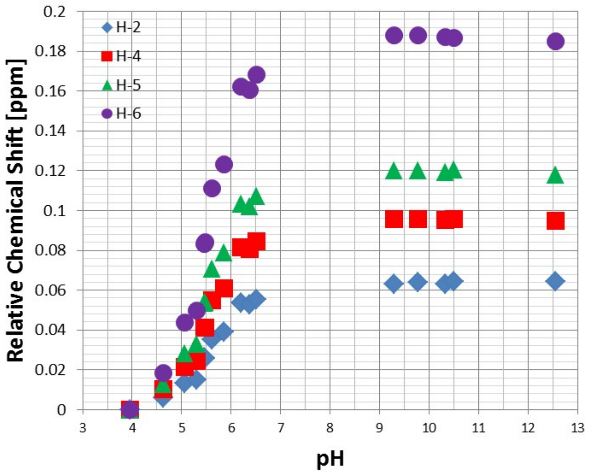

Figure S6. **¹H NMR chemical shifts of free NA as a function of pH in the absence of the catalyst.**

Table S4. **¹H NMR chemical shifts of the indicated resonances of 4 and free NA as a function of pH. e denotes equatorially bound protons while a denotes axially bound protons.**

pH	H-2	H-2e	H-2a	H-4	H-4e	H-4a	H-5	H-5e	H-5a	H-6	H-6e	H-6a
3.63	9.083	9.052	9.017	8.338	8.192	7.699	7.468	7.07	7.048	8.568	8.308	8.078
4.6	9.081	9.050	9.020	8.337	8.191	7.694	7.467	7.071	7.052	8.566	8.411	8.078
5.13	9.083	9.051	9.033	8.337	8.192	7.686	7.467	7.067	7.05	8.566	8.411	8.078
5.31	9.081	9.050	9.020	8.337	8.191	7.694	7.467	7.071	7.052	8.566	8.411	8.078
5.35	9.081	9.050	9.039	8.337	8.191	7.681	7.466	7.065	7.049	8.565	8.411	8.077
5.6	9.084	9.046	9.033	8.339	8.194	7.693	7.469	7.067	7.056	8.568	8.411	8.081
5.75	9.088	9.036	8.999	8.346	8.204	7.732	7.478	7.082	7.072	8.582	8.411	8.090
5.93	9.092	9.025	8.967	8.354	8.213	7.772	7.486	7.105	7.076	8.596	8.411	8.098
6.55	9.105	9.002	8.890	8.372	8.235	7.867	7.509	7.152	7.087	8.632	8.411	8.119
6.96	9.113	8.991	8.849	8.382	8.248	7.919	7.522	7.175	7.094	8.652	8.411	8.131
8.14	9.116	8.986	8.831	8.386	8.252	7.939	7.528	7.185	7.097	8.661	8.419	8.134
11.59	9.13	8.971	8.754	8.407	8.273	8.042	7.553	7.223	7.109	8.698	8.458	8.158
11.8	9.128	8.967	8.740	8.406	8.276	8.053	7.553	7.229	7.109	8.702	8.461	8.159
12.15	9.135	8.955	8.685	8.416	8.292	8.124	7.568	7.256	7.12	8.723	8.484	8.176
11.78	9.141	8.945	8.629	8.425	8.31	8.204	7.579	7.287	7.132	8.743	8.507	8.200
12.08	9.143	8.936	8.582	8.433	8.343	8.241	7.588	7.325	7.152	8.755	8.53	8.284

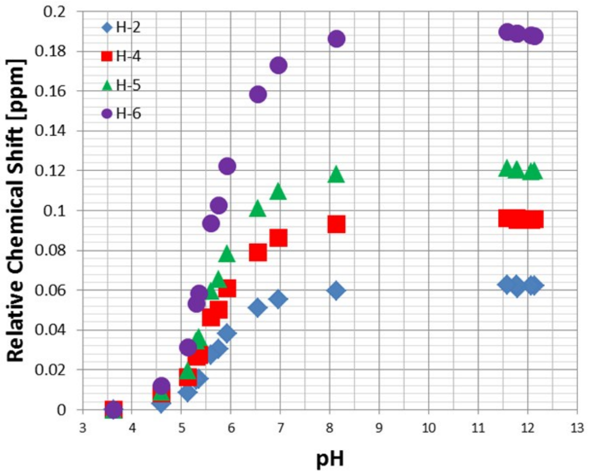

Figure S7. ^{1}H NMR chemical shifts for the indicated protons of NA as a function of pH.

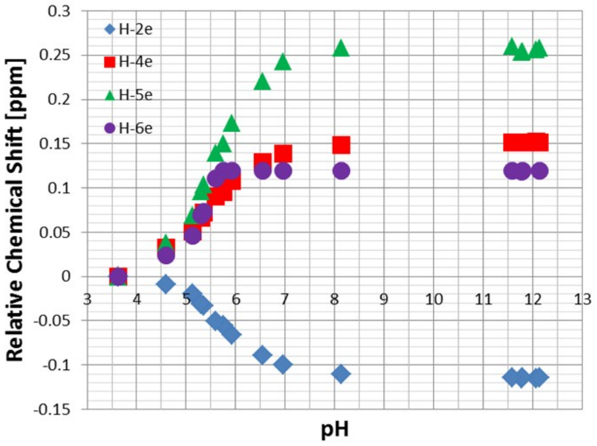

Figure S8. ^{1}H NMR chemical shifts for the indicated equatorially bound protons of NA as a function of pH.

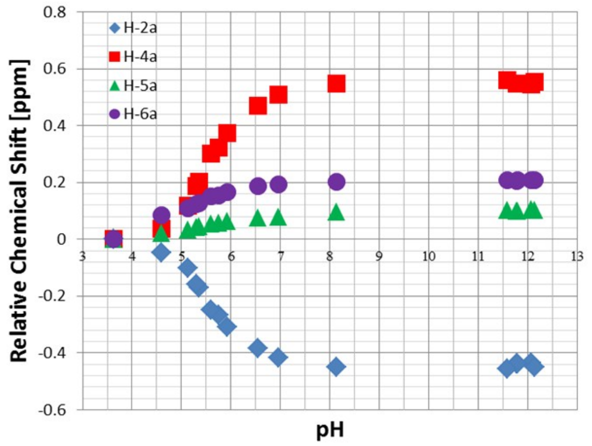

Figure S9. ^{1}H NMR chemical shifts for the indicated axially bound protons of NA as a function of pH.

It is noteworthy that the parent complexes also feature distinctive proton resonances for the hydride ligand, the imidazolium proton and the IMes aromatic CH group at δ –22.70, 7.15 and 6.65 at pH 3.6. In contrast, the corresponding signals at pH 12.5 appear at δ –22.64, 7.07, 6.63.

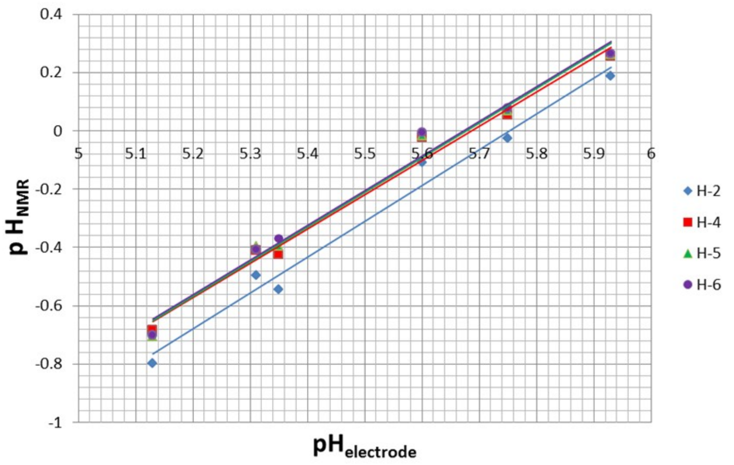

Figure S10. Henderson-Hasselbach plots for the indicated resonances of unbound NA.

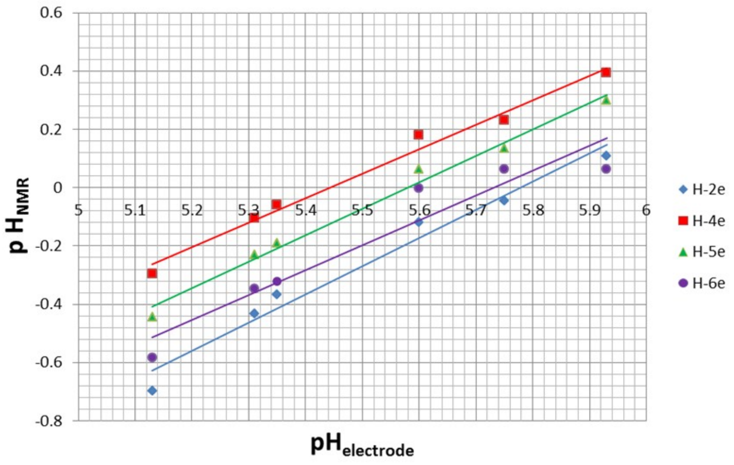

Figure S11. Henderson-Hasselbach plots for the indicated equatorial resonances of NA in 4.

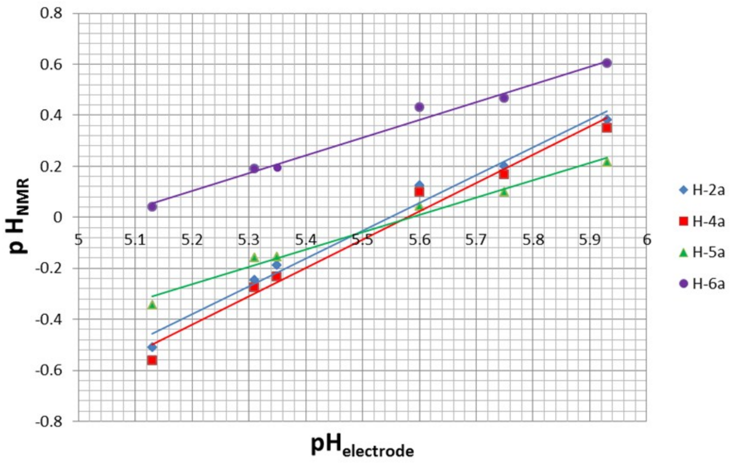

Figure S12. Henderson-Hasselbach plots for the indicated axial resonances of NA in 4.

2.4 pH influence on SABRE enhancements.

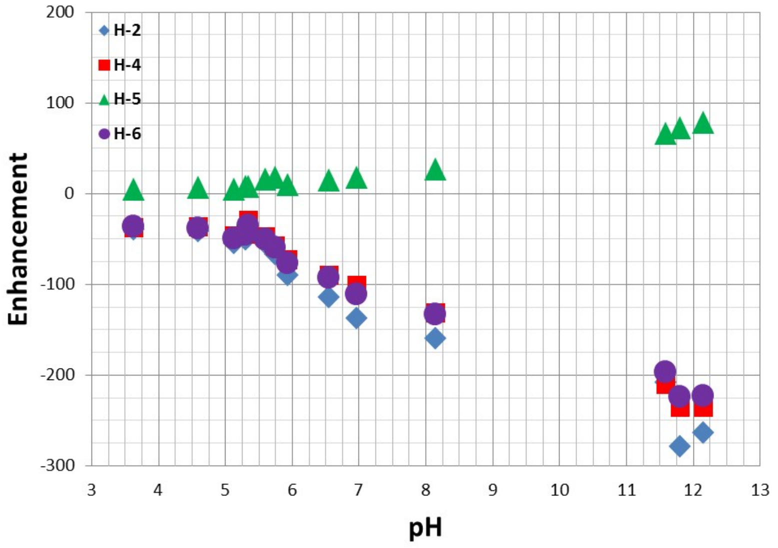

Figure S13. ^{1}H NMR signal enhancements for the indicated free substrate protons as a function of pH.

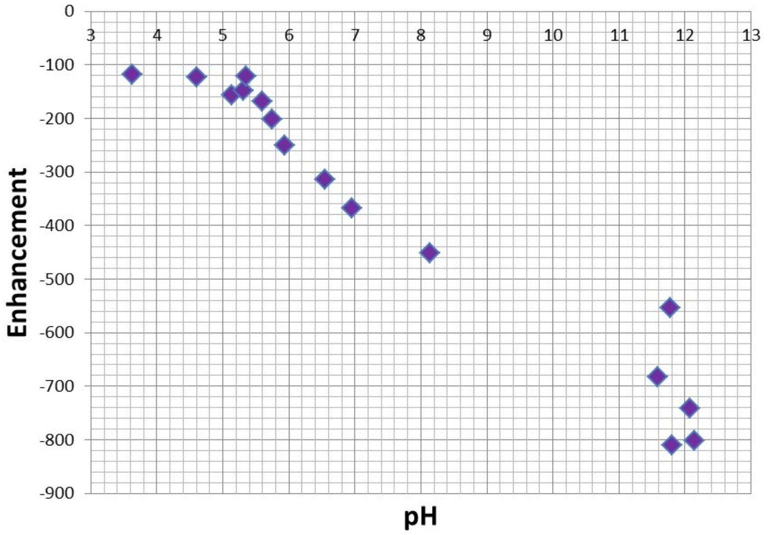

Figure S14. Total signal enhancement measured for the free NA protons as a function of pH.

2.5 Base loading effect on ligand build-up rates in the presence of 4b.

Table S5. Rates of NA build-up in solution as a function of base and substrate excess.

Substrate excess [-fold]	Base excess [-fold]	k_{4b} [1/s] A→B	k_{4b} [1/s] B→A
**1**	**1**	0.93 ± 0.01	0.91 ± 0.03
**3**	**3**	1.40 ± 0.01	0.83 ± 0.02
**5**	**5**	1.41 ± 0.01	0.60 ± 0.02
**7**	**7**	1.48 ± 0.01	0.45 ± 0.01
**9**	**9**	1.62 ± 0.01	0.55 ± 0.01
**13**	**13**	1.83 ± 0.01	0.36 ± 0.01
**17**	**17**	1.78 ± 0.10	0.30 ± 0.02

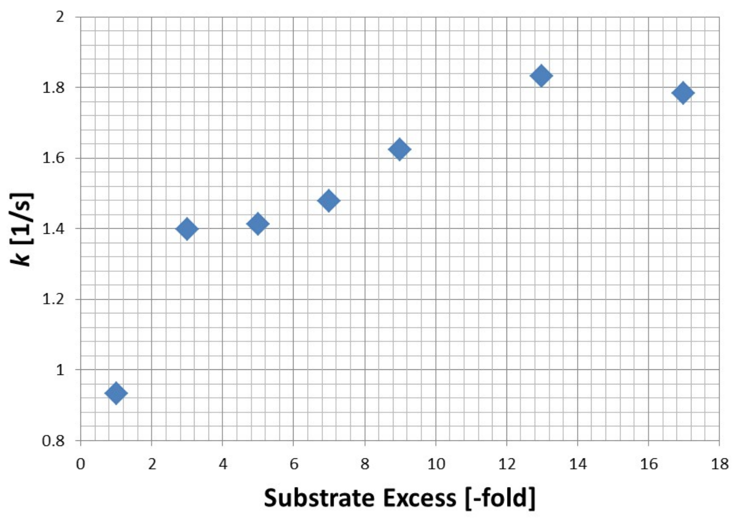

Figure S15. Ligand build-up rates as a function of substrate and base excess.

2.6 Ligand build-up rates in the presence of 4a and 4b as a function of temperature.

Table S6. Rates of ligand build-up as a function of temperature.

Temperature [K]	k_{4a} [1/s]	k_{4b} [1/s]
265	0.06 ± 0.01	0.04 ± 0.01
270	1.12 ± 0.01	0.05 ± 0.01
275	0.26 ± 0.01	0.10 ± 0.01
280	0.52 ± 0.01	0.22 ± 0.01
285	0.99 ± 0.01	0.47 ± 0.01
290	2.01 ± 0.01	0.85 ± 0.02
295	3.79 ± 0.03	1.53 ± 0.01
300	6.99 ± 0.07	3.33 ± 0.01
305	12.96 ± 0.20	5.40 ± 0.09
310	24.74 ± 0.62	8.46 ± 0.29
315	32.73 ± 0.74	-

Table S7. Activation parameters for NA build-up from 4a and 4b respectively.

Activation Parameters	4a	4b
$\Delta H^{\neq}$ (kJ mol^{-1})	88	88
+/-	2.2	3.4
$\Delta S^{\neq}$ (J K^{-1} mol^{-1})	70.4	64.4
+/-	7.9	11.9
$\Delta G^{\neq}_{300}$ (kJ mol^{-1})	66.9	69.4
+/-	0.4	0.3
R Square	0.998	0.998

2.7 Effect of polarisation transfer field on the level of ^{1}H SABRE enhancement.

Samples with a 17-fold excess of **NA** relative to 4 in methanol-d_4 were polarised using PTF values ranging from 0 to 140 G in 10 G steps. The results obtained for the free ligand single spin enhancement values are shown in Figure S16. Both **4a** and **4b** therefore behave in a similar fashion, with the maximum enhancement level being obtained at around 70 G for H-2 in each case. The highest values are consistently achieved for free **NA**'s ortho protons, H-2 and H-6, as they are closest to the binding site.

Whilst H-2, H-4 and H-6 yield single maxima, H-5 exhibits a more complex behaviour, first changing from positive to negative amplitudes up to 70 G and then reaching a second local maximum, at 110 G before increasing again as the PTF reaches 140 G. This behaviour is typical for protons located in the *meta* position, which often experience multiple phase changes as the PTF is varied.[16] It is for this reason that we used a PTF of 45 G in the experiments, even though it does not provide the largest signal gain. It is worth noting that all of these resonances receive a significant increase in polarisation with **4b** in conjunction with the fully deprotonated substrate.

The enhancement ratios for **4b/4a** at 70 G are 5.3, 4.5, 1.9 and 5.5 for H-2, H-4, H-5 and H-6 respectively. Similar or higher increases were observed in the amplitudes of the higher order longitudinal terms that are created under SABRE (see Figures S17-S21). It was established previously that the observe *para*-hydrogen spectroscopy (OPSY) protocol allows such terms to be probed very simply in protio solvents, such as H_2O, without the need for further solvent suppression.[7]

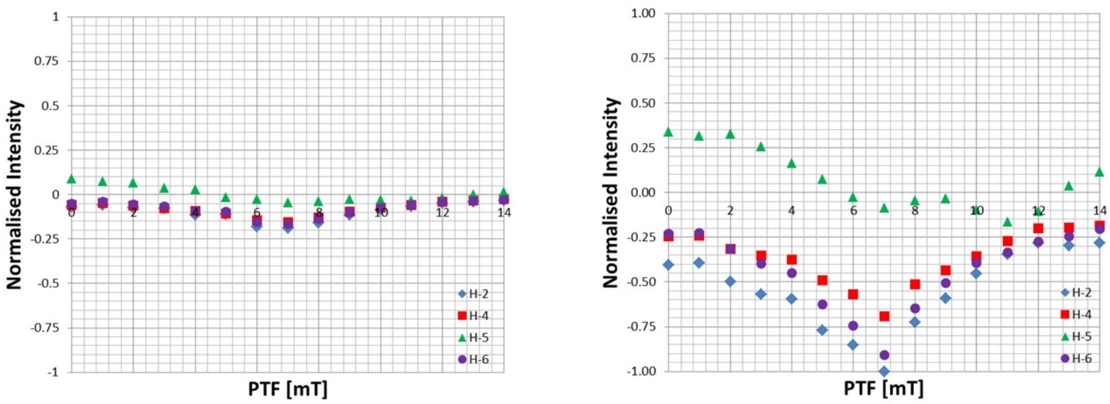

Figure S16. Field dependence plot for 4a (left) and 4b (right). All data have been divided by the absolute value of the highest individual proton intensity (obtained, in this case for H-2 in the presence of 4b). For comparison purposes data are plotted on the same scale.

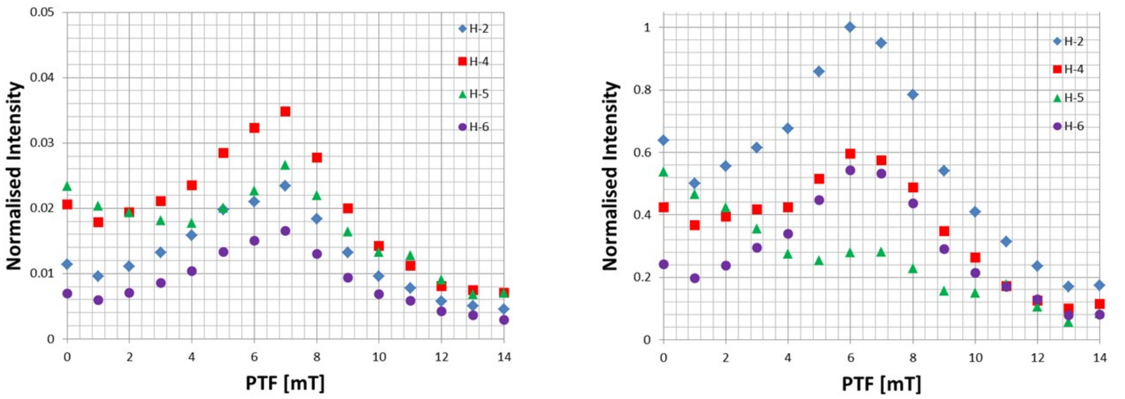

Figure S17. Total zero quantum (ZQ) amplitudes versus PTF for 4a (left) and 4b (right). All data have been divided by the absolute value of the highest individual proton intensity (obtained, in this case for H-2 in the presence of 4b).

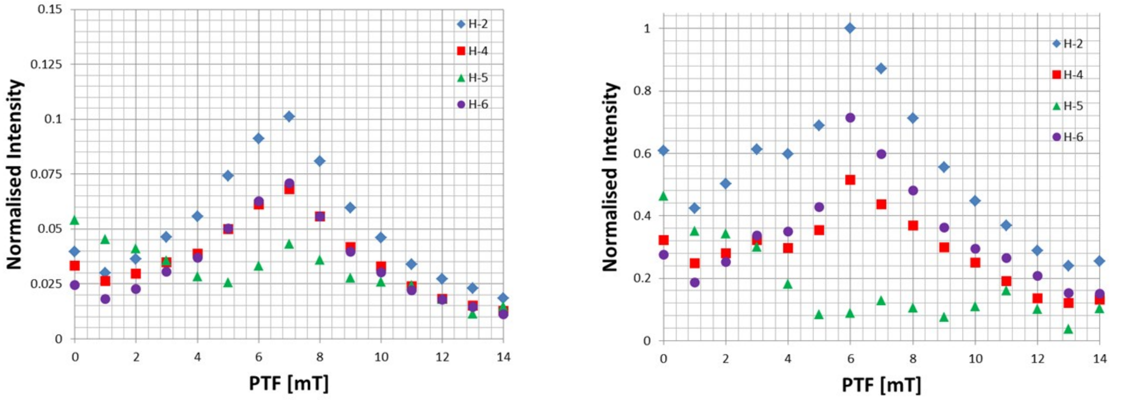

Figure S18. Total single quantum (SQ) amplitudes versus PTF for 4a (left) and 4b (right). All data have been divided by the absolute value of the highest individual proton intensity (obtained, in this case for H-2 in the presence of 4b).

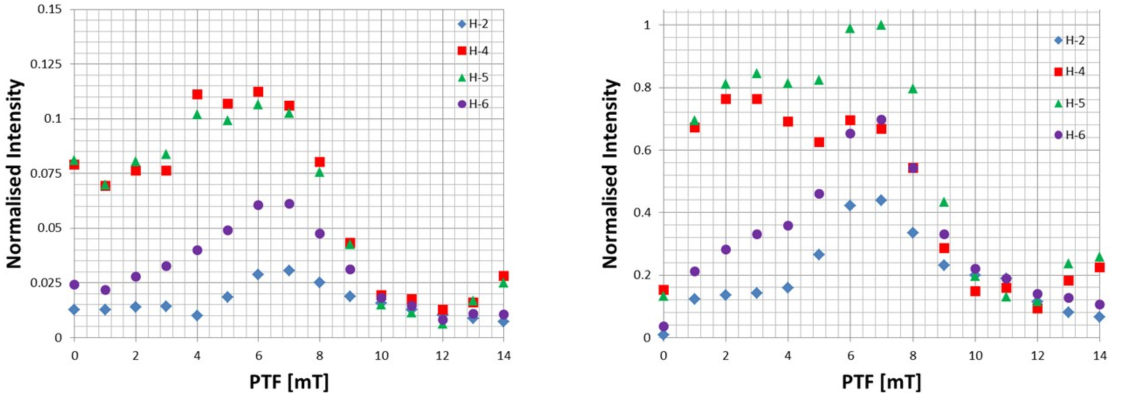

F

igure S19. Total double quantum (DQ) amplitudes versus PTF for 4a (left) and 4b (right) . All data have been divided by

the absolute value of the highest individual proton intensity (obtained, in this case for H-5 in the presence of 4b).

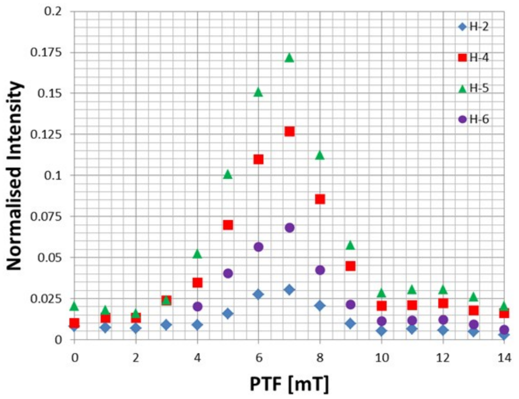

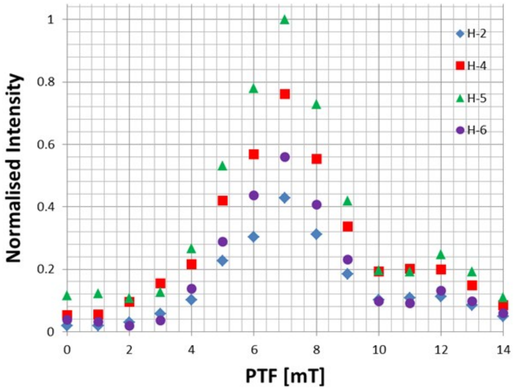

Figure S20. Total triple quantum (TQ) amplitudes versus PTF for 4a (left) and 4b (right). All data have been divided by the absolute value of the highest individual proton intensity (obtained, in this case for H-5 in the presence of 4b).

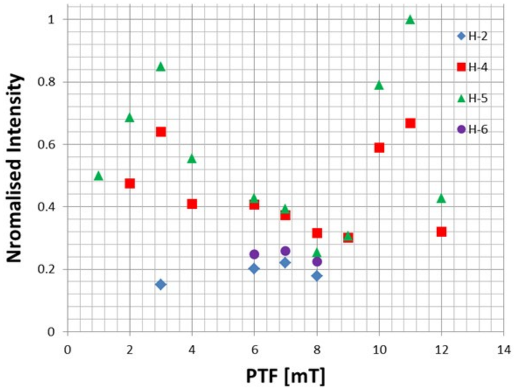

Figure S21. Quadruple quantum (QQ) state amplitude versus PTF for 4b. Data have been normalised to the maximum intensity value of H-5.

We have used the only parahydrogen spectroscopy protocol (OPSY[7]) to selectively observe the signals derived from zero, single, double, triple and quadruple quantum coherences that are derived from the associated longitudinal terms created under SABRE. This approach could allow for the selective observation of **NA** in a fully protio solvent when the ZQ, DQ, TQ or QQ terms are observed.

2.8 Effect of PTF on the level of ^{13}C SABRE signal enhancement of nicotinic acid.

While ^{1}H and ^{15}N benefit from direct and/or indirect polarisation, the ^{13}C resonances are more difficult to detect, as they are effectively polarised only through their couplings with protons. In order to determine the effect of the change on pH has on ^{13}C enhancements, samples containing 17-fold excess nicotinic acid in the presence of **4a** and **4b** have been analysed using the automated system described previously.

While no enhancement could be detected in the presence of **4a**, we found that the addition of base and ensuing pH increase (deprotonation of the nitrogen centre of **NA$_C$**) enables the detection of **NA** ^{13}C signals. ^{13}C polarised NMR spectra obtained for both samples, at a PTF value of 0.5 G, are presented in Figure S22, which shows that base addition facilitates the detection of all **NA** ring ^{13}C resonances.

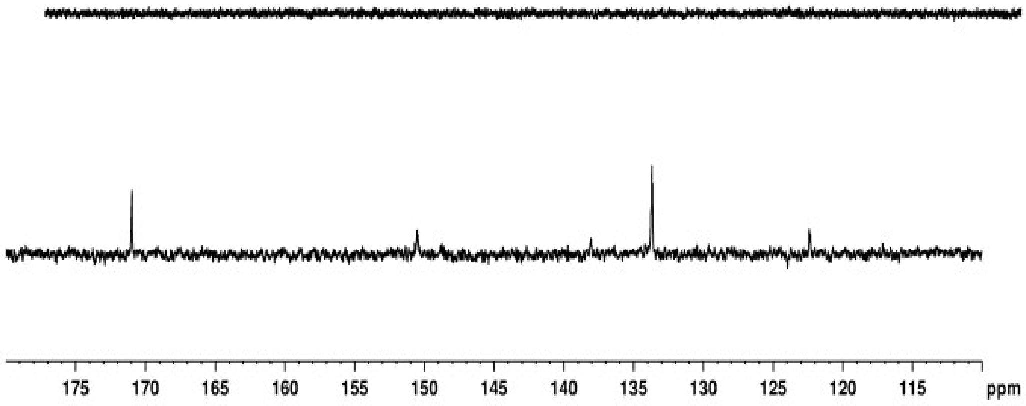

Figure S22. Comparative results obtained for ^{13}C NMR spectra of 4a (top) and 4b (bottom) obtained using 17-fold excess of NA in methanol-*d*$_4$. These NMR spectra were acquired using 32 scans and employed a polarisation transfer field of 0.5 G.

2.9 Effect of PTF on the level of ^{13}C SABRE signal enhancement of ^{13}C labelled nicotinic acid.

In order to analyse in more detail the behaviour of the SABRE enhancements of **NA** as a function of the polarisation transfer field, we prepared samples containing **4a** and **4b** and a 17-fold excess of **NA** whose carboxylic group was ^{13}C enriched. These samples were placed in a μ-metal shield, as described previously, and SABRE enhanced NMR spectra acquired for PTF values ranging from 0 to 1 G, in 0.1 increments (Figures S23 and S24). The associated enhancement values are presented in Figure S25; they were calculated by dividing the integral areas of the polarised peaks to those of appropriate NMR spectra acquired under Boltzmann equilibrium conditions.

For both **4a** and **4b**, the maximum ^{13}C signal enhancement value is obtained using a PTF of 0 G. It can be seen that in the case of **4b**, the enhancement is considerably higher and, unlike **4a**, strong polarisation of the equatorial bound resonance can also be detected in agreement with the slower dissociation rate.

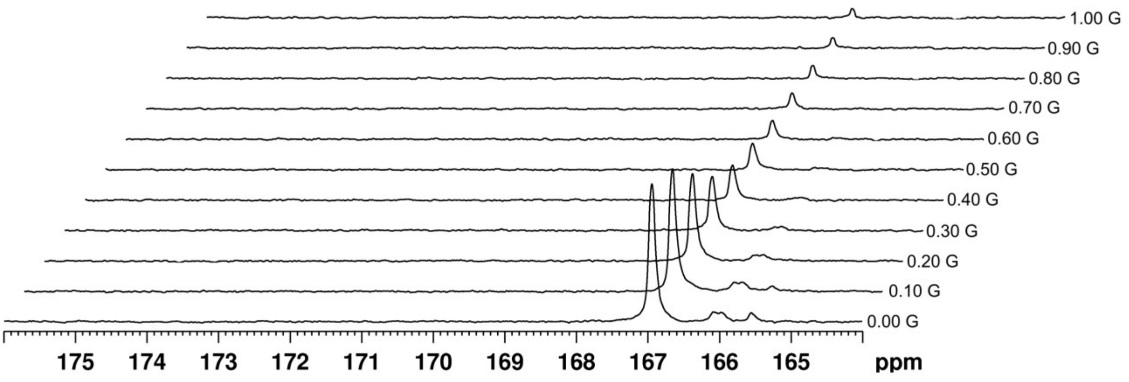

Figure S23. ^{13}C SABRE enhanced spectra resulting from transfer by 4a as a function of the PTF.

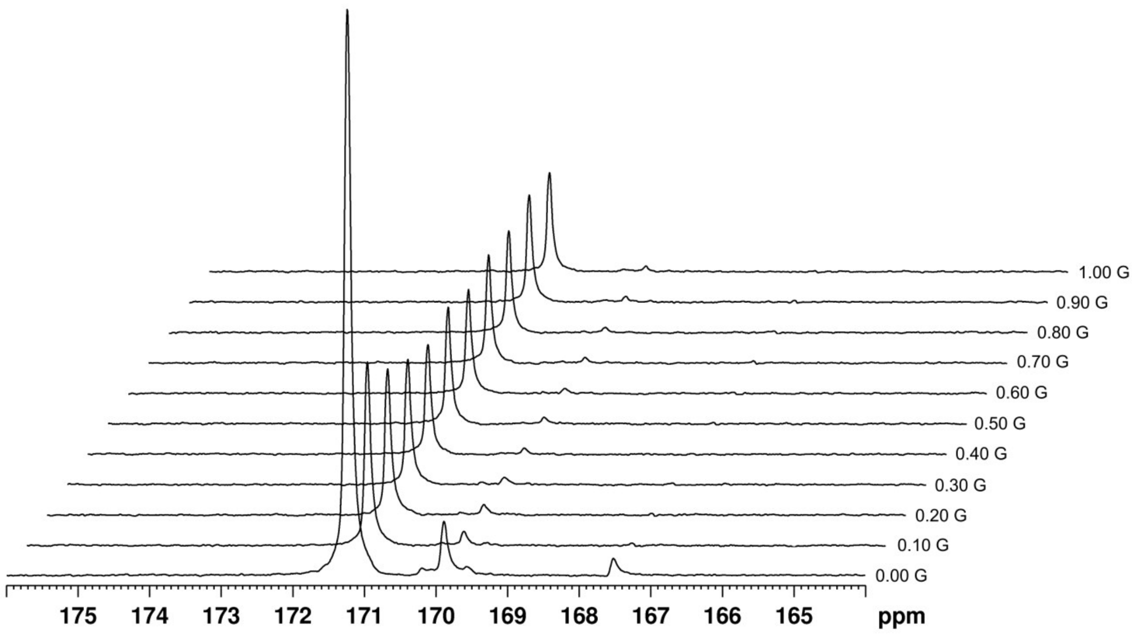

Figure S24. ^{13}C SABRE enhanced spectra resulting from transfer by 4b as a function of the PTF.

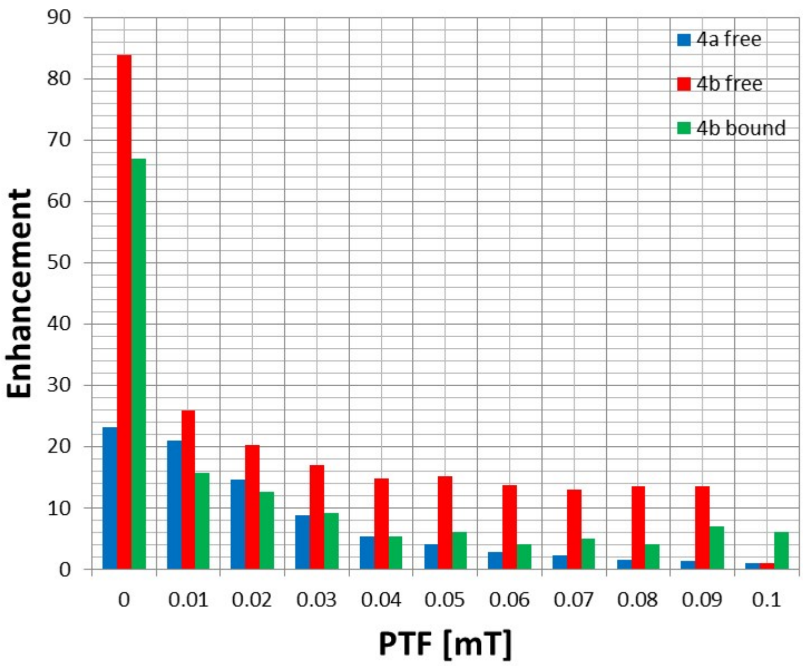

Figure S25. Summary of the signal enhancement values achieved for the ^{13}C carbonyl resonance of NA in the presence of 4a or 4b in methanol-d_4 solution as a function of polarisation transfer field (PTF).

2.10 Effect of PTF on the level of ^{15}N SABRE signal enhancement.

The variation in the level of SABRE observed with PTF was also probed for ^{15}N by placing the sample in a μ-metal shield that screens the effect of the Earth's magnetic field, thereby allowing us to employ discrete values below 0.5 G. Samples containing **4a** and **4b** with 17-fold excess NA were analysed both with and without ^{1}H-decoupling pulses. The results for **4b** (without proton decoupling) are presented in Figure S26, and show that the highest signal enhancement is obtained when employing a PTF of 0.2 G. While the same field value is optimal for **4a** dramatically lower signal enhancement results.

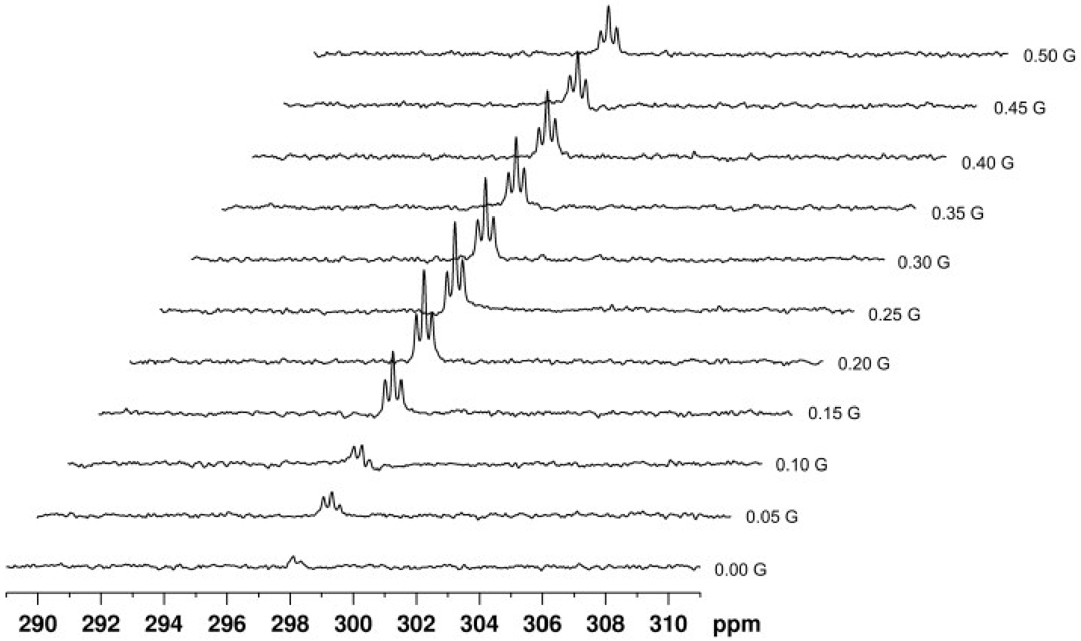

Figure S26. ^{15}N NMR spectra resulting from transfer by 4b as a function of PTF.

These observations again show that the addition of base significantly improves the ^{15}N response. The absolute enhancement for the ^{1}H decoupled ^{15}N resonance of **4b**, calculated via a ^{15}N labelled pyridine standard, was 9218-fold.

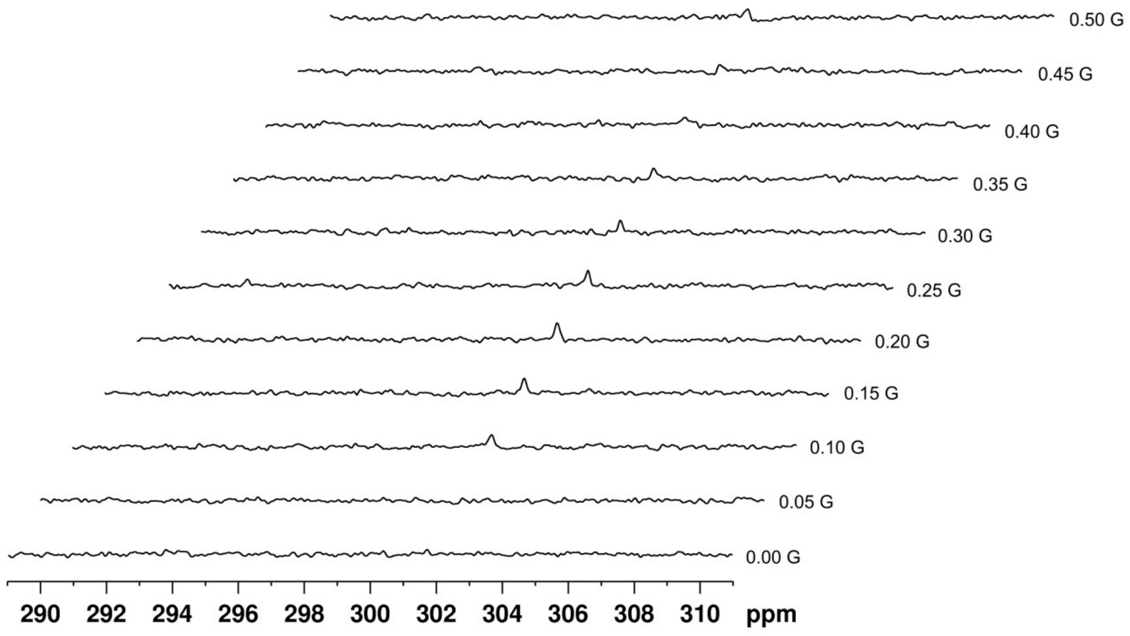

Figure S27. **^{1}H decoupled ^{15}N NMR spectra resulting from transfer by 4a as a function of PTF.**

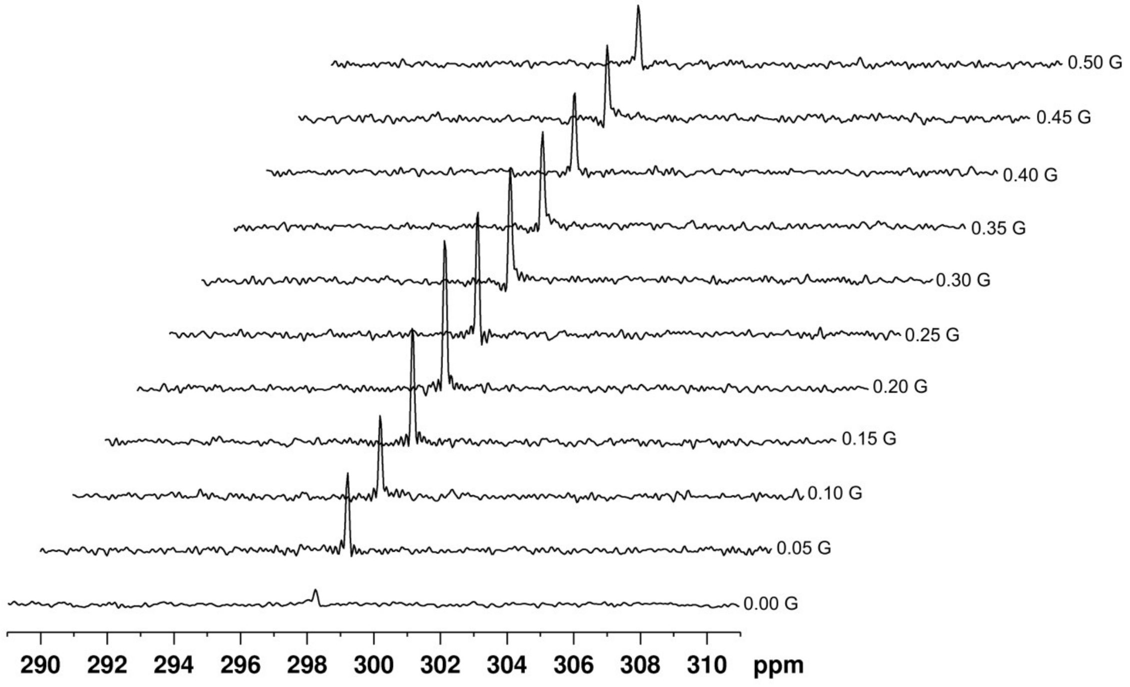

Figure S28. **^{1}H decoupled ^{15}N NMR spectra resulting from transfer by 4b as a function of PTF.**

2.11 pH influence on solvent polarisation

Several reports of methanol participating in the active polarisation transfer catalyst can be found in the literature[17-18] and, as observed by Moreno and co-workers, enhancement of the O**H** proton can be easily attained in mild acidic conditions.[19] As solutions of **4a** in methanol-d_4 have an inherently low pH due to the acidic nature of the substrate, we have analysed the behaviour of the O**H** resonance as a function of substrate excess, pH and PTF in order to rationalise the level of solvent signal enhancement as a function of the polarisation transfer condition.

When analysing these data obtained for **4a**, as the ligand loading is progressively increased, we find that the residual OH resonance of the solvent is considerably enhanced, decreasing with substrate amount from a maximum of approximately 100-fold for up to a 7-fold excess of **NA** to 68-fold for a 17-fold excess of **NA** (see Figure S29). This fall matches with the findings of Lloyd,[18] Moreno,[19] and Eshuis[20] who have shown that at low ligand loadings the solvent competes most effectively for one of the equatorial sites in the polarisation transfer complex.

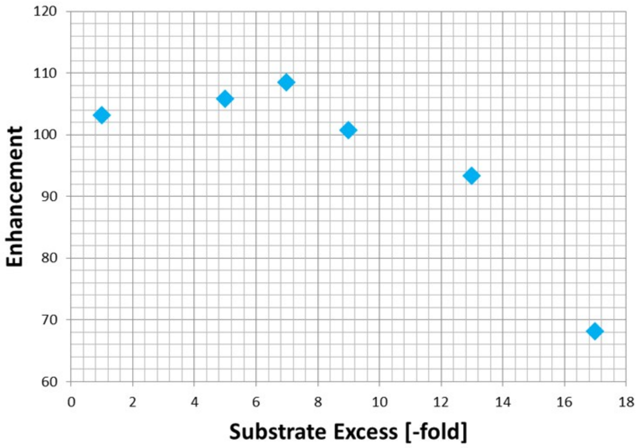

Figure S29. **^{1}H NMR based CD_3-OH signal enhancement as a function of substrate excess.**

This leads to the formation of species exemplified by $[Ir(H)_2(IMes)(MeOH)(ligand)_2]$ and a smaller amount of polarisation transfer to the substrate. As the ligand loading is increased, so does the probability of $[Ir(H_2)(IMes)(ligand)_3]Cl$ being formed and hence less solvent is exposed to the SABRE effect, thereby decreasing the CD_3-O**H** signal gain.

These observations match the trend shown in Figure 2 of the main paper, which shows that the enhancement level found for the **NA** protons increases as the **NA** excess is raised from 1- to 5-fold. When the amount of base added can fully deprotonate the acid, the CD_3-O**H** enhancement decreases suddenly from 70-fold to approximately 3-fold. The effect of the polarisation transfer field on the polarisation of CD_3-O**H** resonance of **4a** has been assessed for values between 0 and 140 G. The results, presented in Figure S30, show that the CD_3-O**H** resonance is significantly enhanced in acidic conditions and that the maximum amount of polarisation is attained at slightly lower fields than for the **NA** resonances. When analysing the corresponding results for **4b** we found that the maximum CD_3-O**H** resonance amplitude is attained at 50 G.

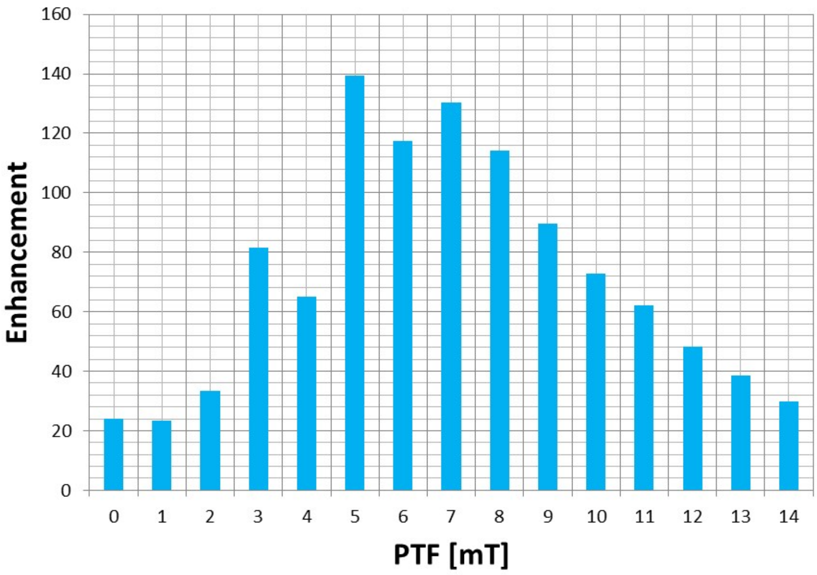

Figure S30. ^{1}H **NMR based** CD_3**-OH signal enhancement as a function of polarisation transfer field under acid conditions where 4a operates.**

2.12 Complex characterisation by NMR.

2.12.1 NMR characterisation data for 2a in methanol-d_4 at 245 K (298 K is indicated with *)

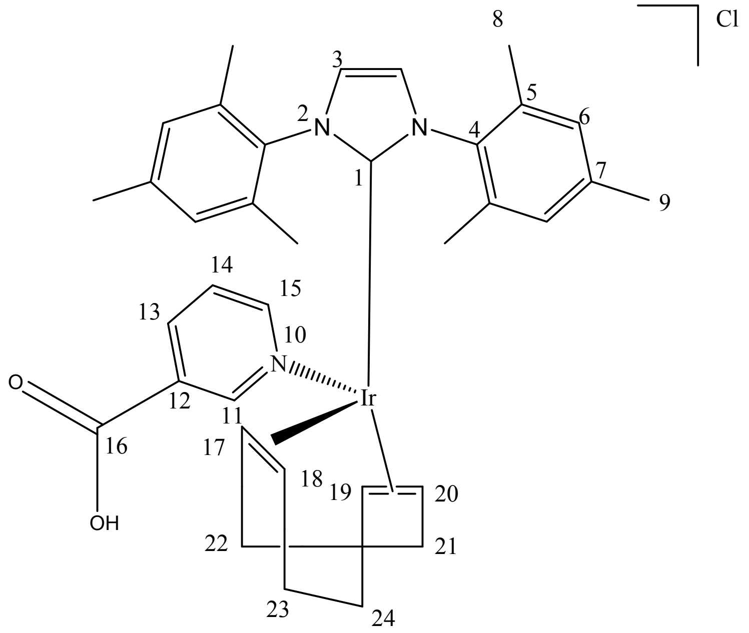

Resonance number	^{1}H (ppm)	^{13}C (ppm)	^{15}N (ppm), free 298.4
**1**		172.2	
**2**			195.8
**3**	7.46 s	125.3	
**4**		135.4	
**5**		138.3*	
**6**	7.23 s	129.2	
**7**		140.05	
**8**	2.38 br	19.7*	
**9**	2.42 s	19.82	
**10**			242.7
**11**	8.24	151.40	
**12**		164.4	
**13**	8.35, d, 8 Hz	137.9	
**14**	7.50, dd, 6, 8 Hz	125.86	
**15**	8.31 d, 6 Hz	153.10	
**16**			
**17-20**	3.36 br		
**21-24**	2.08 br, 1.91 br and 1.70 br		
**17, 18***	3.28	81.9	
**19, 20***	3.74	65.2	
**21, 24***	1.94, 1.69	32.1	
**22, 23***	2.07,1.68	28.8	

2.12.2 NMR characterisation data for 2b in methanol-d_4 at 245 K

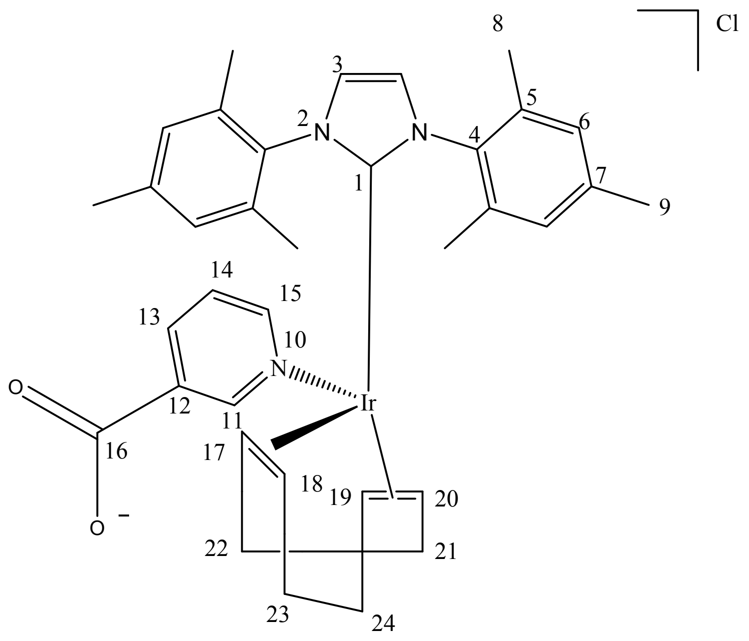

Resonance number	^{1}H (ppm)	^{13}C (ppm)	^{15}N (ppm), free 296.6
**1**		172.5	
**2**			195.7
**3**	7.44 s	125.2	
**4**		119.8	
**5**		135.5	
**6**	7.21 s	129.1	
**7**		140.0	
**8**	2.35 br	17.3	
**9**	2.42 s	19.9	
**10**			240.6
**11**	8.24 d, 2 Hz	151.45	
**12**		168.6	
**13**	8.17, td, 2, 8 Hz	137.3	
**14**	7.37, dd, 6, 8 Hz	125.04	
**15**	8.06 dd, 2, 6 Hz	150.99	
**16**			
**17-20**	3.36 br	81.72	
**21-24**	2.06 br, 1.88 br and 1.66 br		

2.12.3 NMR characterisation data for 3a in methanol-d_4 at 245 K

After adding H_2 to **2a** at 245 K the resonances corresponding to [Ir(H)$_2$(IMes)(**NA$_B$**)]Cl (**3a**) can be detected at δ -11.91 and δ -17.32 (Figure S31).

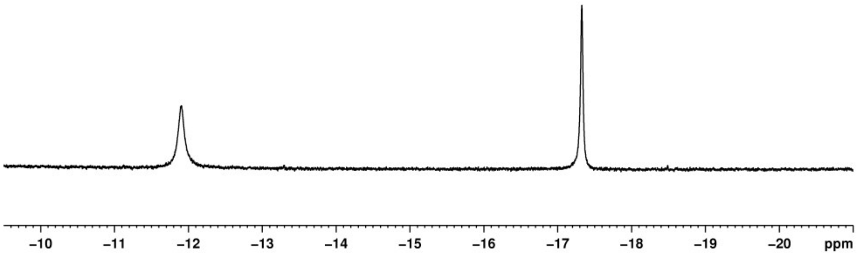

Figure S31. ^{1}H NMR spectrum of the hydride signals of 3a.

The slow activation of the parent complex is observed to form **3a**, and these resonances are then complemented by hydride signals at δ -22.48 (corresponding to [Ir(H)$_2$(IMes)(**NA$_B$**)$_3$]Cl) and δ -21.96, δ -23.0, δ -23.38 and δ -23.45 ppm, as detailed in Figure S32. These signals are indicative of the formation of a pair of further dihydride complexes that contain inequivalent hydride ligands. Furthermore, a pair of broad signals can be detected at δ -24.22 and -27.20, where the chemical are shifts typical for hydrides *cis* and *trans* to MeO**H**, as shown in our previous work (Figure S32).[17] Another single resonance, is present at δ -30.96 ppm that is indicative for the formation of a complex in which the ligand binds through the oxygen atom in the CO***O***$^-$ group, instead of the N centre. When these resonances are examined for PHIP at 245 K, enhancement can be observed for all except the hydride corresponding to **4a** and the upfield resonance at δ -30.96 ppm. Upon warming further to room temperature the resonances of **4a** dominate.

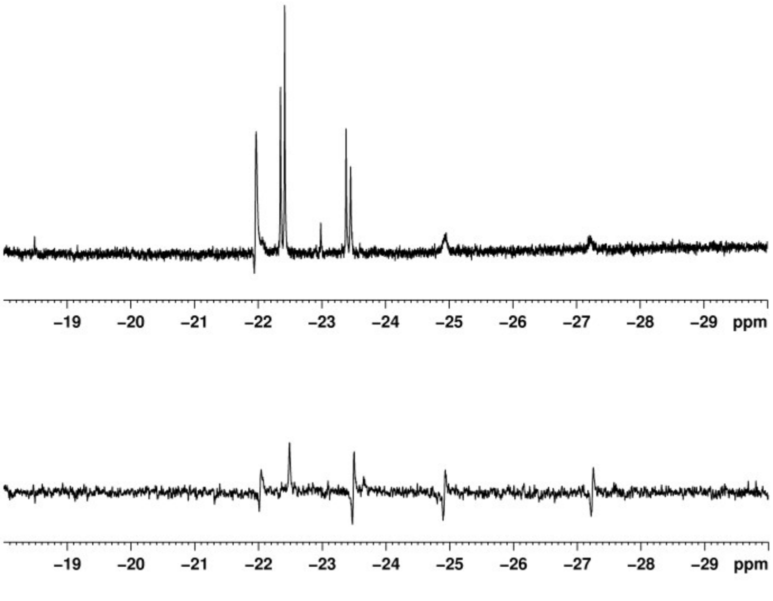

Figure S32. Top: ^{1}H NMR spectrum detailing the downfield hydride ligand region for [Ir(H)$_2$(COD)(IMes)(NA$_B$)]Cl (3a) at 245 K with the signals for 4a being present at -22.48 ppm (NS = 256). Bottom: PHIP enhanced spectrum of the hydride resonances (NS = 1).

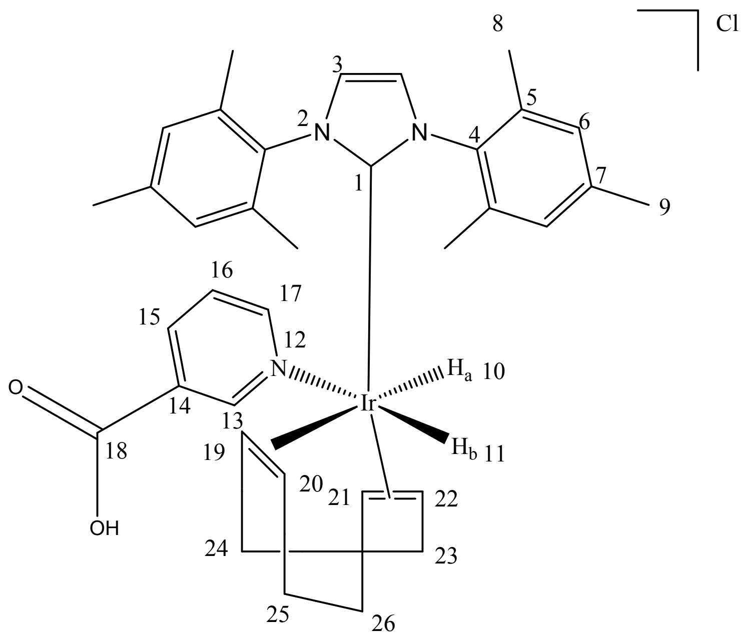

Resonance number	^{1}H (ppm)	^{13}C (ppm)	^{15}N (ppm)
**1**		153.8	
**2**			197.2
**3**	7.38	124.48	
**4**		136.6	
**5**		135.0, 135.26	
**6**	7.08, 6.91	129.0	
**7**		139.71	
**8**	2.02, 1.93	17.01, 16.92	
**9**	2.37	19.86	
**10**	-11.91		
**11**	-17.32		
**12**			255.2
**13**	8.72 s	157.9	
**14**		164.8	
**15**	8.50 overlap	137.64	
**16**	7.43 overlap	126.27	
**17**	8.84 br	156.5	
**18**		-	
**19**	4.77	91.75	
**20**	4.32	88.4	
**21**	3.65	85.0	
**22**	4.99	81.6	
**23**	2.11, 1.93	-	
**24**	2.02, 2.51	-	
**25**	2.08, 1.82	25.16	
**26**	2.28, 1.27	30.21	

2.12.4 NMR characterisation data for 3b in methanol-d_4 at 245 K

After adding H_2 to **2b** at 245 K the resonances corresponding to $[Ir(H)_2(IMes)(\mathbf{NA_D})]Cl$ (**3b**) can be detected at δ -12.18 and δ -17.37 (Figure S33).

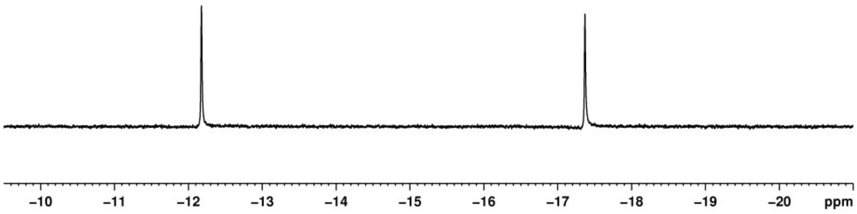

Figure S33. ^{1}H NMR spectrum detailing the downfield hydride ligand region for $[Ir(H)_2(COD)(IMes)(NA_D)]Cl$ (3b).

As for **3a**, partial activation of the complex takes place and the formation of $[Ir(H)_2(COD)(IMes)(NA_D)_3]Cl$ (**4b**) is indicated by a diagnostic resonance at δ -22.36 (Figure S34). However, unlike the spectra acquired after the addition of H_2 to **2a**, which exhibited a complex hydride region due to the formation of several inequivalent complexes, only the trisubstituted complex can be detected in the downfield region of the spectra acquired for **4b**. This shows that the base addition leading to the deprotonation of the nitrogen centre and conversion of $\mathbf{NA_B}$ into $\mathbf{NA_D}$ allows the substrate to bind more efficiently than in the previous case and prohibits the formation of other complexes.

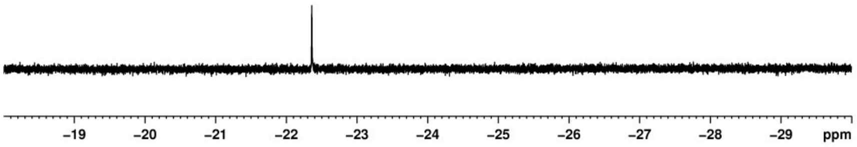

Figure S34. ^{1}H NMR spectrum detailing the downfield hydride ligand region for $[Ir(H)_2(COD)(IMes)(NA_D)]Cl$ (3b) at 245 K with the signal for 4b being present at -22.36 ppm (NS = 128).

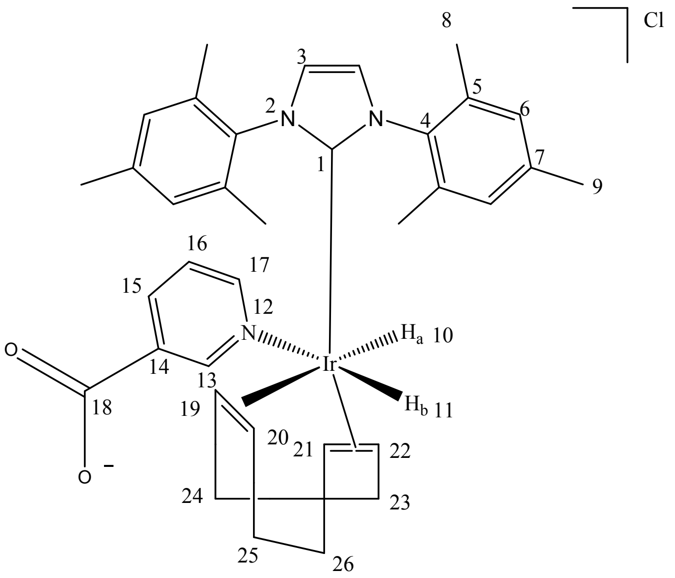

Resonance number	^{1}H (ppm)	^{13}C (ppm)	^{15}N (ppm)
**1**		153.8	
**2**			197.6
**3**	7.38	124.4	
**4**		136.61	
**5**		135.1, 135.0	
**6**	7.04, 6.94	128.94, 129.14	
**7**		139.7	
**8**	1.98, 1.95	17.1	
**9**	2.37	20.0	
**10**	-12.18		
**11**	-17.37		
**12**			218.9
**13**	8.85 s		
**14**		169.0	
**15**	8.34 (overlap)	135.3	
**16**	7.28 dd 8, 6 Hz	125.6	
**17**	8.53 d 6 Hz	136.7	
**18**			
**19**	4.62	92.0	
**20**	4.35	89.3	
**21**	3.69	84.56	
**22**	5.014	80.67	
**23**	2.11	31.96	
**24**	2.29, 1.96	33.83	
**25**	2.07, 1.87	25.78	
**26**	2.14, 1.30	29.63	

2.12.5 NMR characterisation data for 4a in methanol-d_4 at 298 K

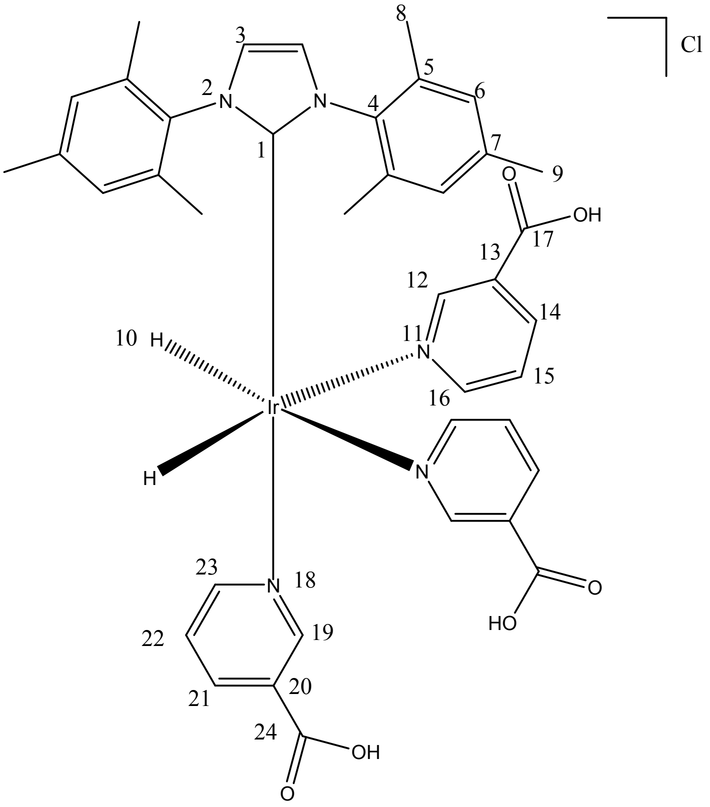

Resonance number	^{1}H (ppm)	^{13}C (ppm)	^{15}N (ppm)
**1**		151.1	
**2**			194.6
**3**	7.15	122.7	
**4**		135.0	
**5**		137.1	
**6**	6.65	128.5	
**7**		133.4	
**8**	2.07	17.5	
**9**	2.15	19.6	
**10**	-22.63		
**11**			255.50
**12**	8.93, s	155.9	
**13**			
**14**	8.34, d, 8 Hz	137.0	
**15**	7.33, dd, 8, 6 Hz	125.2	
**16**	8.53, d, 6 Hz	156.5	
**17**			
**18**			238.3
**19**	8.59, s	156.0	
**20**			
**21**	8.24, d 8 Hz	137.3	
**22**	7.14, d 8, 6 Hz	125.4	
**23**	8.28, d, 6 Hz	158.4	
**24**			

2.12.6 NMR characterisation data for 4b in methanol-d_4 at 298 K

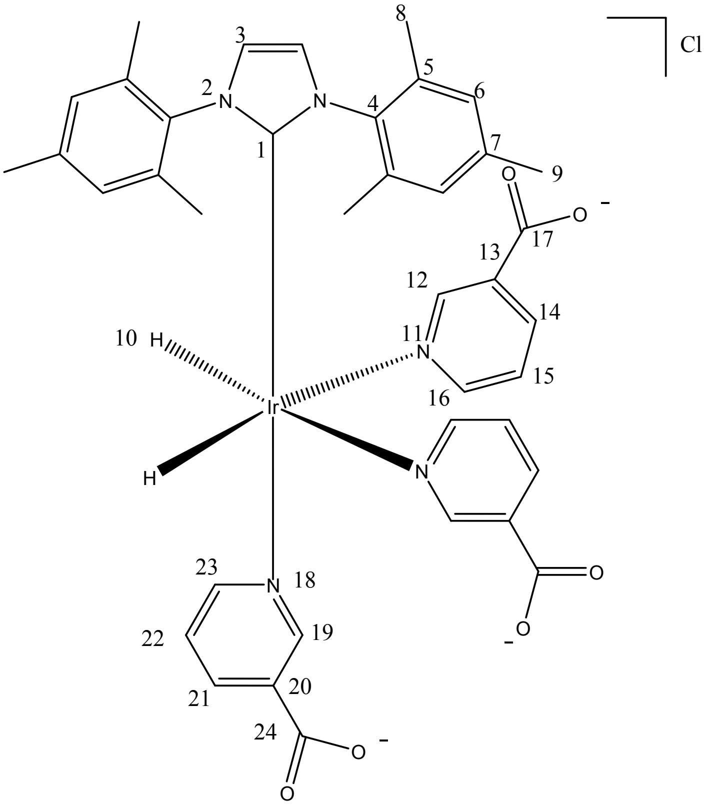

Resonance number	^{1}H (ppm)	^{13}C (ppm)	^{15}N (ppm)
**1**		152.8	
**2**			194.5
**3**	7.065	122.2	
**4**		135.0	
**5**		137.4	
**6**	6.63	128.5	
**7**		138.2	
**8**	2.07	17.67	
**9**	2.17	19.72	
**10**	-22.64		
**11**			254.3
**12**	9.05 s	155.4	
**13**		133.7	
**14**	8.19, d 8 Hz	136.2	
**15**	7.05, dd, overlap	124.0	
**16**	8.3, overlap	154.9	
**17**		173.3	
**18**			237.4
**19**	9.01 s	158.2	
**20**			
**21**	8.08, d 8 Hz	136.3	
**22**	6.87, dd, overlap	124.4	
**23**	7.70, d, 6 Hz	154.2	
**24**			

3. MRI Experimental Procedures and Results

3.1 ^{1}H MRI experiments

All experiments have been performed using a 400 MHz Bruker Avance spectrometer equipped with a microimaging gradient set with maximum amplitude of 1T/m and a double resonance birdcage coil with a diameter of 30 mm.

The ^{1}H MRI acquisition parameters were, as follows: field of view 25 x 25 mm^2, slice thickness 2 mm, matrix size 64 x 64 (zero-filled to 128 x 128) leading to a nominal resolution of 0.39 x 0.39 mm^2 (digital resolution 0.195 x 0.195 mm^2). A sine bell squared filter has been applied prior to the Fourier transform to minimise the contribution of noise in the images. Data post processing has been done using home developed routines in Prospa (Magritek) and MATLAB (Mathworks). All samples have been prepared in 5 mm diameter standard NMR tubes and the hyperpolarisation step has achieved by shaking the sample under *p*-H_2 for 10 seconds at 65G in the stray field of the magnet.

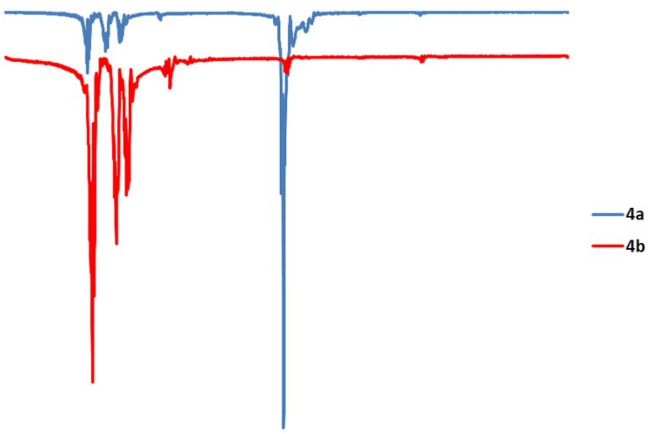

Figure S35. ^{1}H NMR spectra of hyperpolarised NA dissolved in methanol-d_4 with acidic (4a present) and basic (4b present) solutions.

3.2 ^{13}C MRI experiments

The ^{13}C MRI acquisition parameters were, as follows: field of view 30 x 30 mm^2, slice thickness 10 mm, matrix size 64 x 64 (zero-filled to 128 x 128) leading to a nominal resolution of 0.47 x 0.47 mm^2 (digital resolution 0.234 x 0.234 mm^2). The sample has been prepared using 5 mM of **1** and 20 equivalents of **NA** and Cs_2CO_3 (17-fold excess) respectively in 3 ml methanol-d_4. The polarisation transfer step has been achieved using the automated system previously described and a μ-metal shield to shield the sample from the Earth's magnetic field.

4. References

1. Torres, O.; Martín, M.; Sola, E., Labile N-Heterocyclic Carbene Complexes of Iridium. *Organometallics* **2009,** *28* (3), 863-870.
2. Stott, K.; Stonehouse, J.; Keeler, J.; Hwang, T.-L.; Shaka, A. J., Excitation Sculpting in High-Resolution Nuclear Magnetic Resonance Spectroscopy: Application to Selective NOE Experiments. *Journal of the American Chemical Society* **1995,** *117* (14), 4199-4200.
3. Shaw, A. A.; Salaun, C.; Dauphin, J.-F.; Ancian, B., Artifact-Free PFG-Enhanced Double-Quantum-Filtered COSY Experiments. *Journal of Magnetic Resonance, Series A* **1996,** *120* (1), 110-115.
4. Ancian, B.; Bourgeois, I.; Dauphin, J.-F.; Shaw, A. A., Artifact-Free Pure Absorption PFG-Enhanced DQF-COSY Spectra Including a Gradient Pulse in the Evolution Period. *Journal of Magnetic Resonance* **1997,** *125* (2), 348-354.
5. Kessler, H.; Oschkinat, H.; Griesinger, C.; Bermel, W., Transformation of homonuclear two-dimensional NMR techniques into one-dimensional techniques using Gaussian pulses. *Journal of Magnetic Resonance (1969)* **1986,** *70* (1), 106-133.
6. Mewis, R. E.; Atkinson, K. D.; Cowley, M. J.; Duckett, S. B.; Green, G. G. R.; Green, R. A.; Highton, L. A. R.; Kilgour, D.; Lloyd, L. S.; Lohman, J. A. B.; Williamson, D. C., Probing signal amplification by reversible exchange using an NMR flow system. *Magnetic Resonance in Chemistry* **2014,** *52* (7), 358-369.
7. Aguilar, J. A.; Elliott, P. I. P.; Lopez-Serrano, J.; Adams, R. W.; Duckett, S. B., Only para-hydrogen spectroscopy (OPSY), a technique for the selective observation of para-hydrogen enhanced NMR signals. *Chemical Communications* **2007,** (11), 1183-1185.
8. Vassilev, N. G.; Dimitrov, V. S., Dynamic NMR: combined use of 1D selective EXSY and complete lineshape analysis of spectra subjected to reference deconvolution and linear prediction or the maximum entropy method. *Magnetic Resonance in Chemistry* **2001,** *39* (10), 607-614.
9. Jones, W. D.; Rosini, G. P.; Maguire, J. A., Photochemical C−H Activation and Ligand Exchange Reactions of CpRe(PPh3)2H2. Phosphine Dissociation Is Not Involved. *Organometallics* **1999,** *18* (9), 1754-1760.
10. Kutta, W., Beitrag zur näherungsweisen Integration totaler Differentialgleichungen. *Z.Math.Phys.* **1901,** *46*, 435-453.
11. Runge, C., Ueber die numerische Auflösung von Differentialgleichungen. *Mathematische Annalen* **1895,** *46* (2), 167-178.
12. Levenberg, K., A Method for the Solution of Certain Non-Linear Problems in Least Squares. *The Quarterly of Applied Mathematics* **1944,** (2), 164-168.
13. Marquardt, D., An Algorithm for Least-Squares Estimation of Nonlinear Parameters. *Journal of the Society for Industrial and Applied Mathematics* **1963,** *11* (2), 431-441.
14. Polanyi, J. C., Concepts in reaction dynamics. *Accounts of Chemical Research* **1972,** *5* (5), 161-168.
15. Eyring, H., The Activated Complex and the Absolute Rate of Chemical Reactions. *Chemical Reviews* **1935,** *17* (1), 65-77.
16. Green, R. A.; Adams, R. W.; Duckett, S. B.; Mewis, R. E.; Williamson, D. C.; Green, G. G. R., The theory and practice of hyperpolarization in magnetic resonance using parahydrogen. *Progress In Nuclear Magnetic Resonance Spectroscopy* **2012,** *67*, 1-48.
17. Fekete, M.; Bayfield, O.; Duckett, S. B.; Hart, S.; Mewis, R. E.; Pridmore, N.; Rayner, P. J.; Whitwood, A., Iridium(III) Hydrido N-Heterocyclic Carbene–Phosphine Complexes as Catalysts in Magnetization Transfer Reactions. *Inorganic Chemistry* **2013,** *52* (23), 13453-13461.
18. Lloyd, L. S.; Asghar, A.; Burns, M. J.; Charlton, A.; Coombes, S.; Cowley, M. J.; Dear, G. J.; Duckett, S. B.; Genov, G. R.; Green, G. G. R.; Highton, L. A. R.; Hooper, A. J. J.; Khan, M.; Khazal, I. G.; Lewis, R. J.; Mewis, R. E.; Roberts, A. D.; Ruddlesden, A. J., Hyperpolarisation through reversible interactions with parahydrogen. *Catalysis Science & Technology* **2014,** *4* (10), 3544-3554.

19. Moreno, K. X.; Nasr, K.; Milne, M.; Sherry, A. D.; Goux, W. J., Nuclear spin hyperpolarization of the solvent using signal amplification by reversible exchange (SABRE). *Journal of Magnetic Resonance* **2015,** *257*, 15-23.
20. Eshuis, N.; Hermkens, N.; van Weerdenburg, B. J. A.; Feiters, M. C.; Rutjes, F. P. J. T.; Wijmenga, S. S.; Tessari, M., Toward Nanomolar Detection by NMR Through SABRE Hyperpolarization. *Journal of the American Chemical Society* **2014,** *136* (7), 2695-2698.
21. Gallagher, F. A.; Kettunen, M. I.; Day, S. E.; Hu, D.-E.; Ardenkjaer-Larsen, J. H.; Zandt, R. i. t.; Jensen, P. R.; Karlsson, M.; Golman, K.; Lerche, M. H.; Brindle, K. M., Magnetic resonance imaging of pH in vivo using hyperpolarized 13C-labelled bicarbonate. *Nature* **2008,** *453* (7197), 940-943.
